# Subsets of preoperative sex hormones in testicular germ cell cancer: a retrospective multicenter study

**DOI:** 10.1038/s41598-023-41915-7

**Published:** 2023-09-05

**Authors:** Péter Törzsök, David Oswald, Klaus-Peter Dieckmann, Markus Angerer, Lukas Christian Scherer, Piotr Tymoszuk, Yannic Kunz, Germar-Michael Pinggera, Lukas Lusuardi, Wolfgang Horninger, Renate Pichler

**Affiliations:** 1 Department of Urology and Andrology, Paracelsus Medical University Salzburg, Salzburg, Austria; 2 Department of Urology, Hodentumorzentrum, Asklepios Klinik Altona, Hamburg, Germany; 3 Department of Urology, Comprehensive Cancer Center Innsbruck (CCCI), Medical University of Innsbruck, Anichstrasse 35, 6020 Innsbruck, Austria; 4 Data Analytics As a Service Tirol, Innsbruck, Austria

**Keywords:** Testicular cancer, Gonadal disorders

## Abstract

Preoperative homeostasis of sex hormones in testicular germ cell tumor (TGCT) patients is scarcely characterized. We aimed to explore regulation of sex hormones and their implications for histopathological parameters and prognosis in TGCT using a data-driven explorative approach. Pre-surgery serum concentrations of luteinizing hormone (LH), follicle-stimulating hormone (FSH), testosterone (T), estradiol (E2) and prolactin were measured in a retrospective multicenter TGCT cohort (n = 518). Clusters of patients were defined by latent class analysis. Clinical, pathologic and survival parameters were compared between the clusters by statistical hypothesis testing, Random Forest modeling and Peto-Peto test. Cancer tissue expression of sex hormone-related genes was explored in the publicly available TCGA cohort (n = 149). We included 354 patients with pure seminoma and 164 patients with non-seminomatous germ cell tumors (NSGCT), with a median age of 36 years. Three hormonal clusters were defined: ‘neutral’ (n = 228) with normal sex hormone homeostasis, ‘testicle’ (n = 91) with elevated T and E2, low pituitary hormones, and finally ‘pituitary’ subset (n = 103) with increased FSH and LH paralleled by low-to-normal levels of the gonadal hormones. Relapse-free survival in the hormonal subsets was comparable (p = 0.64). Cancer tissue expression of luteinizing hormone- and follicle-stimulating hormone-coding genes was significantly higher in seminomas, while genes of T and E2 biosynthesis enzymes were strongly upregulated in NSGCT. Substantial percentages of TGCT patients are at increased risk of sex hormone dysfunction at primary diagnosis before orchiectomy. TGCT may directly influence systemic hormonal homeostasis by in-situ synthesis of sex hormones.

## Introduction

Testicular cancer is the most prevalent malignancy in men aged 20–40 years and accounts for one-fourth of tumors in this age group^1^. Testicular germ cell tumors (TGCT) are the most common histopathology^2^. About 55%-60% of the TGCTs are pure seminomas and 40%-45% are non-seminomatous germ cell tumors (NSGCT)^3^. The European Association of Urology define alpha-fetoprotein (AFP), beta subunit of human chorionic gonadotropin (HCG), and lactate dehydrogenase (LDH) as routine serum markers of TGCT^1^. Elevated tumor markers are detected in 15–20% of seminomas and in 60–80% of NSGCT. While HCG positivity is observed in both entities, AFP is restricted to NSGCT^4,5^.

The pre-orchiectomy homeostasis of sex hormones in TGCT is not well characterized. In approximately one-fourth of TGCT patients prior to any kind of therapy, Leydig cell function is impaired, which manifests by lowered serum testosterone (T) as compared with healthy controls^5,6^. Significantly higher systemic estradiol (E2) levels were observed in NSGCT than in seminoma^7–9^. Furthermore, HCG positivity was associated with NSGCT histology, increased pre-surgery levels of E2, and diminished secretion of pituitary sex hormones follicle-stimulating hormone (FSH) and luteinizing hormone (LH)^8^. Hence, concomitant monitoring of T and LH was proposed for evaluation of Leydig cell dysfunction^10^. Pre-operative hormonal abnormalities were associated with increased TGCT burden. Tumor size was reported to correlate with elevated LH and FSH, which may result from disrupted feedback signals from the normal testicular tissue, diminished sperm production and increased E2^6,8^. Leydig cell dysfunction is linked to testicular germ cell neoplasia in situ, advanced age, and a larger tumor size^6,11,12^. Furthermore, the tumor marker HCG displays a substantial structural similarity with LH and a LH-like biological activity, which may contribute to hormonal disorders in HCG-positive patients^13–15^.

Little is known about sex hormone production by the malignant TGCT tissue. Development of NSGCT was associated with polymorphisms of gonadal hormone biosynthesis genes CYP11A1 and HSD17B1^16^. The estrogen excess theory suggests that an imbalance of estrogen can interfere with testicular growth, promote in situ neoplasia and its transition to TGCT^17^. It has been proposed that maternal hormone exposition in utero may affect the risk of seminoma or NSGCT development^16^. Male transgenic mice over-expressing the aromatase (CYP19A1) showed an increased E2 production and a trend to forming Leydig cell tumors^18^.

TGCTs have various E2 receptors that can control seminoma cell proliferation when encountering endocrine disruptors in a laboratory setting. High levels of estrogen may interfere with the normal differentiation and proliferation of germ stem cells during fetal, perinatal, and post-puberty stages^19^. Furthermore, pure seminomas can produce oestrogens without elevation of HCG, which can lead to a hormonal dysbalance resulting in gynaecomastia^14^.

The data on sex hormonal status of patients with TGCT before orchiectomy is not clearly understood. Many studies, dealing with the hormonal status of TGCT patients are only based on postoperative data, which raises the urgent need for a pre-orchiechtomy analysis. We aimed to investigate co-regulation of gonadal and pituitary sex hormones and their implication for pathology and prognosis in TGCT in the largest retrospective multicenter cohort using a data-driven explorative approach. To this end, we assessed the expression of genes involved in sex hormone synthesis in the malignant tissue samples of the TCGA collective^20^.

## Material and methods

### Study design and approval

Patients with histologically confirmed TGCT by inguinal orchiectomy were retrospectively enrolled from three university hospitals during January 2000 and December 2021. The following clinical data were collected for each patient: age, body mass index, tumour size, histology (seminoma/NSGCT), tumour markers, presence of lymphovascular invasion (LVI) or infiltration rete testis, clinical stage, and survival. The exclusion criterion was presence of benign testicular tumors, no data about preoperative serum levels of LH, FSH, E2, T and PRL, and > 50% of missing study data for each patient. A total of n = 518 patients were included in the analysis (Supplementary Fig. S1). Tumor tissue expression of sex-hormone related genes was assessed with normalized mRNA data available for the testicular cancer subset of the pan-cancer TCGA cohort (The Cancer Genome Atlas)^20^.

The retrospective study was conducted in accordance with the Declaration of Helsinki and European Data policy. Participants’ data were stored and analyzed in anonymized form. Consent of the local ethics committees was obtained (Medical University of Innsbruck, approval number 1055/2021; Ethics Committee of the Province of Salzburg, approval number 1106/2022). Informed consent was obtained from all subjects. No ethics committee approval was required for the TCGA data use.

### Procedures

Preoperative serum concentrations of LH, FSH, E2, T (total and free) and PRL were measured with enzyme-linked immunosorbent assays. Reference values were 1.7–8.6 mU/mL for LH, 1.0–10.0 mU/mL for FSH, 20–55 pg/mL for E2, 3.50–9.0 ng/mL for total T and 0–480 µU/mL for PRL according to institutional practice. Positive tumor markers were defined as HCG levels > 2 U/I, AFP > 7 ng/mL and LDH > 250 U/I (> upper limit of norm (ULN)). Histological evaluations of orchidectomy specimens were done by a dedicated genitourinary pathologist at each center. Gene expression data of the TCGA samples represented by batch-normalized mRNA counts and the accompanying clinical information were downloaded from cBioPortal (https://www.cbioportal.org/).

### Follow-up

Due to the retrospective nature of the study the follow-up of TGCT was not standardized. Patients were followed according to contemporary international guidelines, institutional standards based on patient and tumor characteristics^21^. The mean follow-up time was 46.7 months.

### Endpoints

The primary analysis endpoints were investigation of co-regulation of sex hormones prior to orichectomy and definition of subsets of TGCT patients based on pre-operative sex hormone levels. This goal was accomplished by principal component analysis, correspondence analysis and latent class analysis. The secondary analysis endpoints were comparison of histology, demographic and clinicopathological characteristic of the hormonal subsets, comparison of relapse-free survival between the hormonal subsets, and evaluation of mRNA expression of genes involved sex hormone biosynthesis in the TGCT tissue. These tasks were accomplished by statistical hypothesis testing, Peto-Peto test for differences in survival and multi-parameter Random Forest modeling.

### Statistical analysis

Details on statistical analysis are provided in Supplementary Material. Statistical analysis was done with R version 4.2.3.

#### Descriptive statistic and statistical hypothesis testing

Numeric variables are presented as medians with interquartile ranges, ranges and number of complete observations. Categorical variables are presented as percentages and counts within the complete observation set. P values were corrected for multiple testing with the false discovery rate method^22^. Effects with p < 0.05 are considered statistically significant. Normality of numeric variables was assessed by Shapiro–Wilk test and quantile–quantile plots. Since normality assumption was deeply violated for multiple numeric variables, non-parametric statistical hypothesis tests were used. Differences between histological types were investigated with Mann–Whitney test with r effect size metric or χ^2^ test with Cramer V effect size statistic. Differences between hormonal subsets were investigated by Kruskal–Wallis test with η^2^ effect size statistic or χ^2^ test with Cramer V effect size statistic, as appropriate. Relapse-free survival was compared between the histology types or hormonal subsets with Peto-Peto test.

#### Co-regulation of sex hormones, definition of the hormonal subsets

These analyses were done for n = 422 retrospective cohort subjects with complete hormone data. Co-regulation of sex hormones was investigated by four-dimensional principal component analysis (normalized, median-centered hormone concentrations)^23^ and two-dimensional correspondence analysis (hormone levels stratified by reference range limits)^24^.

Hormonal subsets of the retrospective cohort patients were defined by latent class analysis (poLCA R package)^25,26^ of hormone levels stratified by their reference range limits. The optimal number of hormonal subsets (k = 3) was motivated by the minimal value of Bayesian Information Criterion suggestive of the optimal model fit in a comparison of latent class analysis models with varying numbers of classes.

#### Multi-parameter modeling of the hormonal subsets

A multi-parameter model allowing for discrimination between the hormone subsets with solely non-endocrine explanatory variables (available observations: n = 305) was established with the conditional Random Forest algorithm (R packages party and caret, mtry = 18, number of trees = 500)^27–29^. Model performance was assessed in the training dataset and tenfold cross-validation by the accuracy and Cohen’s κ statistic. Accuracy of assignment for particular hormonal subsets was assessed by inspection of heat maps of the confusion matrices.

#### Expression of sex hormone-related genes in the TCGA cohort

Expression of 14 sex hormone-related genes in the tumor samples of the TCGA cohort was investigated (*GNRH1*, *GNRH2*, *PRL*, *CGA*, *FSHB*, *LHB*, *CYP11A1*, *CYP17A1*, *HSD17B3*, *HSD3B1*, *HSD3B2*, *CYP19A1*, *HSD17B1*, *SHBG*). Differences in log_2_-transformed mRNA counts between seminoma and NSGCT were investigated by Mann–Whitney test with r effect size statistic.

## Results

### Study cohort

The study cohort consisted of 518 TGCT patients with a median age of 36 (range: 17–86) years (Supplementary Fig. S1). Pure seminomas were diagnosed in 354 (68%) patients, 164 (32%) cancers were NSGCT. The median tumor size was 3 cm (range: 0.3–15) and 82% (n = 413) of cancers were classified as clinical stage I according to Lugano. Rete testis invasion and lymphovascular invasion (LVI) was detected in 37% and 30% of available TGCT specimens, respectively. AFP positivity rate was 24%, elevated HCG was found in 35% of patients. In general, AFP or HCG above the reference range was detected in 43% of patients. Retroperitoneal lymphadenectomy was performed in 35 (7.8%) patients. Rates of postoperative adjuvant chemotherapy and radiotherapy were 50% and 4%, respectively (Table 1).

**Table 1:** Demographic and cancer-related characteristics of the study cohort.

Variable^a^	Statistic
Age at surgery, years	36 [IQR: 29–44] Range: 17–86 Complete: n = 518
Body mass index, kg/m^2^	25 [IQR: 23–28] Range: 17–41 Complete: n = 339
Body weight class	Normal: 49% (166) Overweight: 36% (121) Obesity: 15% (52) Complete: n = 339
Tumor size, cm	3 [IQR: 1.7–4.5] Range: 0.3–15 Complete: n = 452
Tumor stage (pT)	pT1: 66% (303) pT2: 29% (131) pT3: 4.8% (22) Complete: n = 456
Lugano classification	1: 82% (413) 2: 14% (73) 3: 4% (20) Complete: n = 506
IGCCCG classification	Good: 95% (232) Intermediate: 3.7% (9) Poor: 1.2% (3) Complete: n = 244
Rete testis infiltration	37% (166) Complete: n = 450
Lymphovascular invasion	30% (135) Complete: n = 454
Histology	Seminoma: 68% (354) NSGCT: 32% (164) Complete: n = 518
AFP, ng/mL	3 [IQR: 2–6.5] Range: 0.7–24,000 Complete: n = 515
AFP strata	0–7 ng/mL: 76% (393) > 7 ng/mL: 24% (122) Complete: n = 515
HCG, IU/L	1.2 [IQR: 1–6.9] Range: 0.2–18,000 Complete: n = 515
HCG strata	0–2 IU/L: 65% (333) > 2 IU/L: 35% (182) Complete: n = 515
LDH, U/L	200 [IQR: 170–250] Range: 3.1–3300 Complete: n = 503
LDH strata	0–250 U/L: 74% (373) > 250 U/L: 26% (130) Complete: n = 503
Tumour markers	AFP/HCG-: 57% (295) AFP/HCG + : 43% (220) Complete: n = 515
Retroperitoneal lymphadenectomy	7.8% (35) Complete: n = 449
Chemotherapy	50% (225) Complete: n = 448
Radiation	4% (18) Complete: n = 447
Postoperative TRT	8.6% (35) Complete: n = 409

Numeric variables are presented as medians with interquartile ranges (IQR) and ranges. Categorical variables are presented as percentages and counts within the complete observation set.

^a^*IGCCCG* International Germ Cell Cancer Collaborative Group, *AFP* alpha fetoprotein, *LDH* lactate dehydrogenase; *HCG* human chorionic gonadotropin, *TRT* testosterone replacement therapy.

### Demographic, pathologic, histological and prognostic characteristics of seminoma and NSGCT

**Figure 1:**
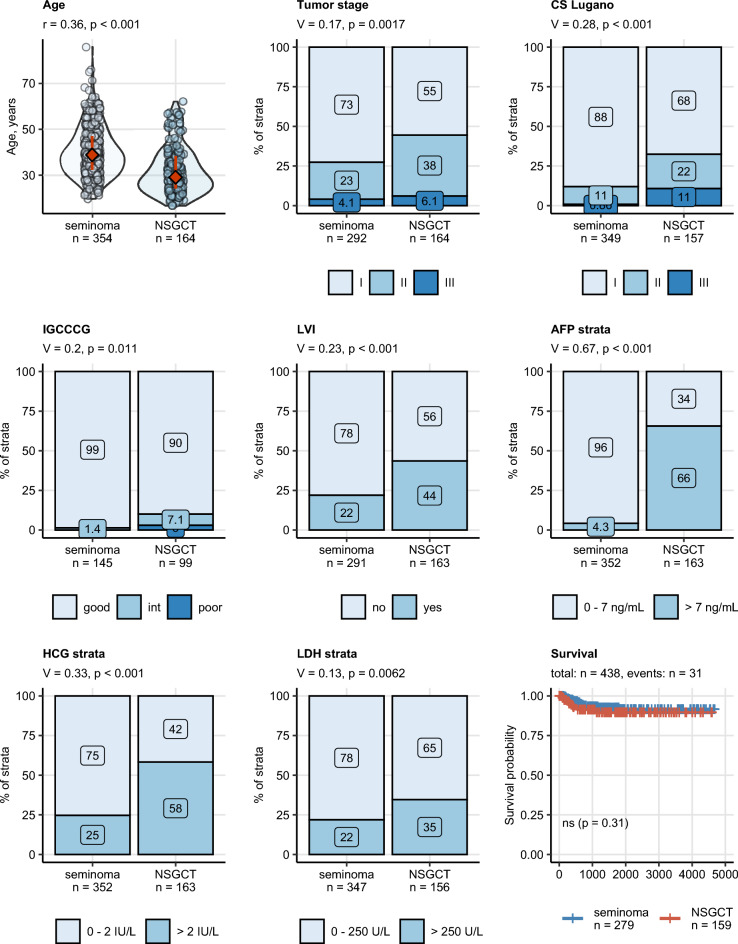
Comparison of age, pathological and clinical stage, IGCCCG stage, lymphovascular invasion, AFP, HCG, LDH strata and relapse-free survival between seminoma and NSGCT. Differences in age at cancer surgery, in blood concentrations of alpha fetoprotein (AFP), human chorionic gonadotropin (HCG) and lactate dehydrogenase (LDH) between the histology types were assessed by Mann–Whitney test with r effect size statistic. Differences in distribution of tumor stages, Lugano classes (CS Lugano), IGCCCG (International Germ Cell Cancer Collaborative Group) risk classes, frequency of LVI, AFP, HCG and LDH strata between the histology types were investigated by χ^2^ test with Cramer V effect size statistic. *P* values were corrected for multiple testing with the false discovery rate (FDR). Numeric variables are presented in violin plots with single observations depicted as points, and medians with interquartile ranges represented by red diamonds and whiskers. Frequencies for levels of categorical variables are shown in stack plots. Effect sizes and *p* values are displayed in the plot captions. Numbers of complete observations are indicated in the X axes. Differences in relapse-free survival between seminoma and NSGCT histology cancers were assessed by log-rank test. Fractions of surviving individuals are presented in a Kaplan–Meier plot. Numbers of observations and relapses are displayed in the plot caption. The test *p* value is shown in the plot.

A number of clinicopathological features differed significantly between seminoma and NSGCT patients (Table 2). In detail, NSGCTs were characterized by a significantly lower onset age, higher tumor stage and IGCCCG risk group as well as more frequent LVI as compared with pure seminoma patients. During postoperative follow-up, we recorded the need of a testosterone replacement therapy (TRT) in 8.6% of patients, while at the time of primary diagnosis, none of the patients received a TRT. Concerning tumor markers, NSGCT patients had also significantly higher levels of AFP (strong difference), HCG (moderate difference) and LDH (weak difference). There were no significant differences in relapse-free survival between seminoma and NSGCT (Fig. 1, Supplementary Fig. S2).

**Table 2:** Demographic and cancer-related characteristics of study participants with seminomas and non-seminomatous germ cell tumors (NSGCT).

Variable^a^	Seminoma	NSGCT	Significance^b^	Effect size^b^
Age at surgery, years	39 [IQR: 32–47] Range: 20–86 Complete: n = 354	29 [IQR: 24–38] Range: 17–62 Complete: n = 164	p < 0.001	r = 0.36
Body mass index, kg/m^2^	25 [IQR: 23–28] Range: 18–40 Complete: n = 211	25 [IQR: 23–28] Range: 17–41 Complete: n = 128	ns (p = 0.12)	r = 0.09
Body weight class	Normal: 46% (98) Overweight: 38% (81) Obesity: 15% (32) Complete: n = 211	Normal: 53% (68) Overweight: 31% (40) Obesity: 16% (20) Complete: n = 128	ns (p = 0.43)	V = 0.074
Tumor size, cm	3 [IQR: 1.5–4.7] Range: 0.3–15 Complete: n = 288	3 [IQR: 2–4.5] Range: 0.7–12 Complete: n = 164	ns (p = 0.5)	r = 0.034
Tumor stage	pT1: 73% (212) pT2: 23% (68) pT3: 4.1% (12) Complete: n = 292	pT1: 55% (91) pT2: 38% (63) pT3: 6.1% (10) Complete: n = 164	p = 0.0017	V = 0.17
Lugano classification	1: 88% (307) 2: 11% (39) 3: 0.86% (3) Complete: n = 349	1: 68% (106) 2: 22% (34) 3: 11% (17) Complete: n = 157	p < 0.001	V = 0.28
IGCCCG risk	Good: 99% (143) Intermediate: 1.4% (2) Poor: 0% (0) Complete: n = 145	Good: 90% (89) Intermediate: 7.1% (7) Poor: 3% (3) Complete: n = 99	p = 0.011	V = 0.2
Rete testis infiltration	40% (117) Complete: n = 289	30% (49) Complete: n = 161	ns (p = 0.059)	V = 0.1
Lymphovascular invasion	22% (64) Complete: n = 291	44% (71) Complete: n = 163	p < 0.001	V = 0.23
AFP, ng/mL	2.4 [IQR: 1.8–3.6] Range: 0.7–15 Complete: n = 352	28 [IQR: 4.6–110] Range: 1–24,000 Complete: n = 163	p < 0.001	r = 0.6
AFP strata	0–7 ng/mL: 96% (337) > 7 ng/mL: 4.3% (15) Complete: n = 352	0–7 ng/mL: 34% (56) > 7 ng/mL: 66% (107) Complete: n = 163	p < 0.001	V = 0.67
HCG, IU/L	1 [IQR: 1–2] Range: 0.3–13,000 Complete: n = 352	7 [IQR: 1–90] Range: 0.2–18,000 Complete: n = 163	p < 0.001	r = 0.29
HCG strata	0–2 IU/L: 75% (265) > 2 IU/L: 25% (87) Complete: n = 352	0–2 IU/L: 42% (68) > 2 IU/L: 58% (95) Complete: n = 163	p < 0.001	V = 0.33
LDH, U/L	200 [IQR: 170–240] Range: 100–3300 Complete: n = 347	220 [IQR: 180–280] Range: 3.1–1600 Complete: n = 156	p = 0.017	r = 0.11
LDH strata	0–250 U/L: 78% (271) > 250 U/L: 22% (76) Complete: n = 347	0–250 U/L: 65% (102) > 250 U/L: 35% (54) Complete: n = 156	p = 0.0062	V = 0.13
Marker status	AFP/HCG-: 72% (254) AFP/HCG + : 28% (98) Complete: n = 352	AFP/HCG-: 25% (41) AFP/HCG + : 75% (122) Complete: n = 163	p < 0.001	V = 0.44
Retroperitoneal lymphadenectomy	0% (0) complete: n = 288	22% (35) complete: n = 161	p < 0.001	V = 0.39
Chemotherapy	40% (116) Complete: n = 287	68% (109) Complete: n = 161	p < 0.001	V = 0.26
Radiation	5.9% (17) Complete: n = 286	0.62% (1) Complete: n = 161	p = 0.018	V = 0.13
Postoperative TRT	9.2% (24) Complete: n = 261	7.4% (11) Complete: n = 148	ns (p = 0.69)	V = 0.03

Numeric variables are presented as medians with interquartile ranges (IQR) and ranges. Categorical variables are presented as percentages and counts within the complete observation set.

^a^*IGCCCG* International Germ Cell Cancer Collaborative Group, *AFP* alpha fetoprotein, *LDH* lactate dehydrogenase, *HCG* human chorionic gonadotropin, *TRT* testosterone replacement therapy.

^b^Numeric variables: Mann–Whitney test with r effect size statistic. Categorical variables: χ^2^ test with Cramer V effect size statistic. P values corrected for multiple testing with the false discovery rate method.

### Pre-orchiectomy sex hormone levels

Decreased total T concentrations (≤ 3.5 ng/mL) were observed in 27% of participants, while supraphysiological levels exceeding the reference range (> 9 ng/mL) were present in 8.6%. Lowered and increased E2 concentrations were found in 32% and 16% of patients, respectively. Most patients (94%) had normal blood levels of PRL, with values above the reference range in only 5.8% of individuals. In 20% of participants FSH levels below the normal range (≤ 1 mU/mL) were present. Abnormally high FSH concentrations (> 10 mU/mL) were observed in approximately one of four patients. Lowered LH was observed in 27% (≤ 1.7 mU/mL) and increased LH concentrations were detected in 9.4% of patients (Table 3).

**Table 3:** Preoperative levels of sex hormones in the study cohort.

Variables^a^	Statistic
Total testosterone, ng/mL	4.4 [IQR: 3.3–6] Range: 1–17 Complete: n = 452
Total testosterone strata	0–3.5 ng/mL: 27% (122) 3.5–9 ng/mL: 64% (291) > 9 ng/mL: 8.6% (39) Complete: n = 452
Free testosterone, ng/mL	10 [IQR: 6.9–14] Range: 0.066–60 Complete: n = 135
E2, pg/mL	28 [IQR: 18–41] Range: 0.5–200 Complete: n = 425
E2 strata	0–20 pg/mL: 32% (134) 20–55 pg/mL: 53% (224) > 55 pg/mL: 16% (67) Complete: n = 425
SHBG, nmol/L	32 [IQR: 22–43] Range: 6.3–160 Complete: n = 47
PRL, µU/mL	170 [IQR: 100–250] Range: 0–4400 Complete: n = 448
PRL strata	0–480 µU/mL: 94% (422) > 480 µU/mL: 5.8% (26) Complete: n = 448
FSH, mU/mL	5.4 [IQR: 2.1–10] Range: 0–100 Complete: n = 447
FSH strata	0–1 mU/mL: 20% (88) 1–10 mU/mL: 55% (245) > 10 mU/mL: 26% (114) Complete: n = 447
LH, mU/mL	3.3 [IQR: 1.6–5.5] Range: 0–47 Complete: n = 449
LH strata	0–1.7 mU/mL: 27% (122) 1.7–8.6 mU/mL: 63% (285) > 8.6 mU/mL: 9.4% (42) Complete: n = 449

Numeric variables are presented as medians with interquartile ranges (IQR) and ranges. Categorical variables are presented as percentages and counts within the eligible observation set.

^a^*T* testosterone; *E2* estradiol, *SHBG* sex hormone binding globulin, *PRL* prolactin, *FSH* follicle-stimulating hormone, *LH* luteinizing hormone.

**Figure 2:**
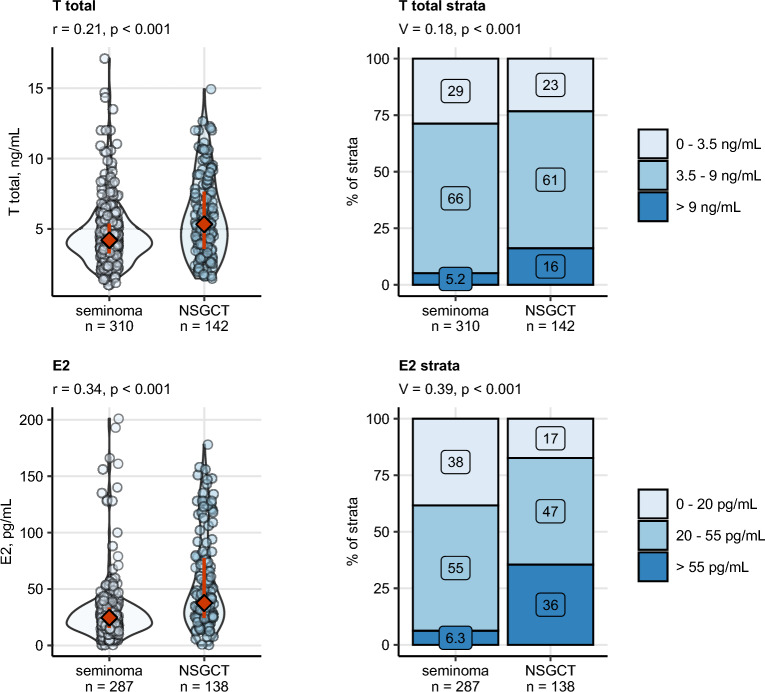
Differences in preoperative levels of total testosterone (T total) and estradiol (E2) between seminoma and non-seminomatous germ cell tumors (NSGCT). Differences in blood concentrations of testosterone (T) and estradiol (E2) between the histology types were assessed by Mann–Whitney test with r effect size statistic. Differences in frequency of clinical strata of hormone levels were investigated by χ^2^ test with Cramer V effect size statistic. P values were corrected for multiple testing with the false discovery rate (FDR) method. Numeric variables are presented in violin plots with single observations depicted as points, and medians with interquartile ranges represented by red diamonds and whiskers. Frequencies for levels of categorical variables are shown in stack plots. Effect sizes and p values are displayed in the plot captions. Numbers of complete observations are indicated in the X axes.

**Figure 3:**
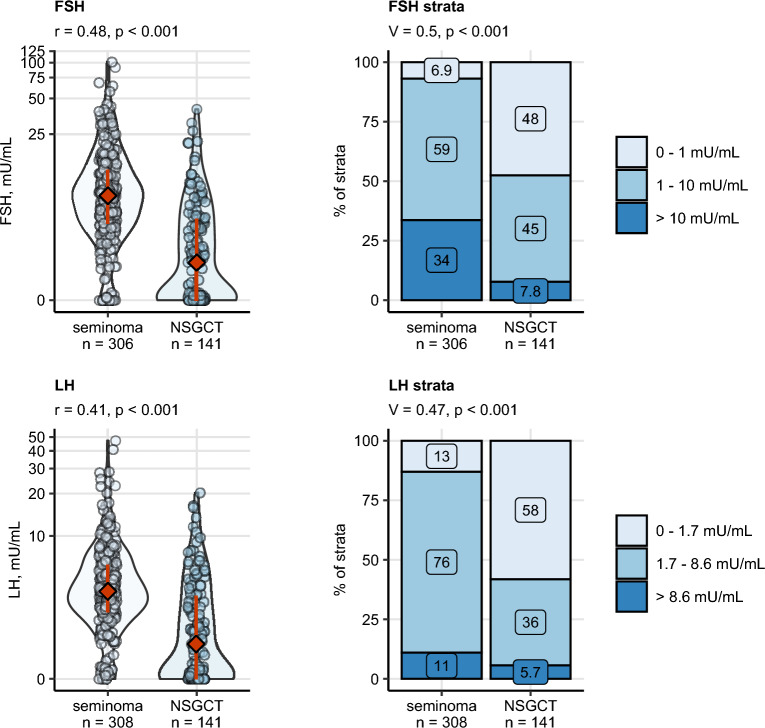
Differences in preoperative levels of follicle-stimulating hormone, luteinizing hormone between seminoma and non-seminomatous germ cell tumors (NSGCT). Differences in blood concentrations of follicle-stimulating hormone (FSH) and luteinizing hormone (LH) between the histology types were assessed by Mann–Whitney test with r effect size statistic. Differences in frequency of clinical strata of hormone levels were investigated by χ2 test with Cramer V effect size statistic. P values were corrected for multiple testing with the false discovery rate (FDR) method. Numeric variables are presented in violin plots with single observations depicted as points, and medians with interquartile ranges represented by red diamonds and whiskers. Frequencies for levels of categorical variables are shown in stack plots. Effect sizes and p values are displayed in the plot captions. Numbers of complete observations are indicated in the X axes.

Total T and E2 concentrations prior to orchiectomy were significantly higher in NSGCT than in seminoma with weak and moderate effect sizes, respectively (Fig. 2). Serum levels of the pituitary sex hormones FSH and LH were moderately lowered in NSGCT as compared with seminoma. In particular, in approximately half of NSGCT patients, suppressed level of FSH or LH was observed as compared with largely normal or elevated FSH and LH in seminoma patients. Furthermore, blood PRL was moderately increased in NSGCT, yet its levels stayed largely within the reference range (Fig. 3).

### Co-regulation of sex hormones

Inspection of the first two major principal components of the hormone dataset (76% of total variance) pointed toward a tight co-regulation of the gonadal hormones (E2 and T) and a substantial inter-correlation of the pituitary hormone levels (LH, FSH and PRL). Additionally, an opposite regulation of the pituitary and gonadal hormones can be inferred (Supplementary Fig. S3A). This interpretation was further corroborated by correspondence analysis performed with hormone levels stratified by their normal range limits. This analysis revealed three modes of patterns of sex hormone regulation in TGCT. Patients with supra-physiological T (> 9 ng/mL) and E2 concentrations (> 55 pg/ml) are expected to have lowered LH and FSH (FSH: ≤ 1 mU/mL, LH: ≤ 1.7 mU/mL) (Supplementary Fig. S3B, left hand side of the plot). In turn, elevated LH (> 8.6 mU/mL) and FSH (> 10 mU/mL) co-coincided frequently with decreased level of gonad hormones (T: ≤ 3.5 ng/mL, E2: ≤ 20 pg/mL) (Suppl. Fig. S2B, top right quarter of the plot). Finally, physiological E2 and T levels were frequently paralleled by normal concentrations of LH and FSH (Supplementary Fig. S3B, bottom right quarter of the plot). Collectively, these results point towards two co-regulation patterns: of the gonad hormones and of the pituitary hormones. These two hormone groups interact in an antagonistic manner, i. e. a hyperactivity of the gonadal axis leading to a suppression of the pituitary axis and the insufficiency of the gonadal axis leading to a hyperactivity of the pituitary axis.

### Definition of sex hormonal subsets in TGCT

**Figure 4:**
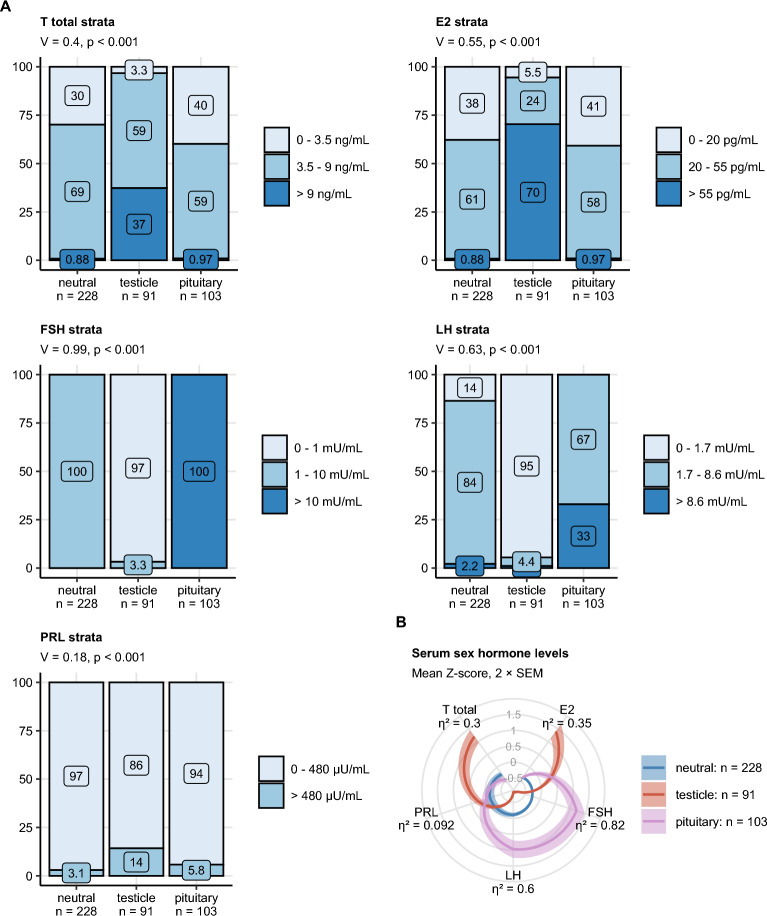
Hormonal subsets of testis cancer patients defined by latent class analysis in respect to preoperative strata of sex hormones and pre-surgery blood concentrations of sex hormones in the hormonal subsets of TGCT. (**A**) Distribution of clinical strata of pre-surgery sex hormone levels in the hormone subsets of testicle cancer defined by latent class analysis (T: testosterone, E2: estradiol, FSH: follicle stimulating hormone, LH: luteinizing hormone, PRL: prolactin). Statistical significance was determined by χ^2^ test with Cramer V effect size statistic. (**B**) Normalized pre-surgery blood concentrations of sex hormones (Z-scores) were compared between the hormonal subsets of testicle cancer defined by latent class analysis by Kruskal–Wallis test with η2 effect size statistic. P values were corrected for multiple testing with the false discovery rate method. Strata frequencies within the hormonal subsets are presented in stack plots. Effect sizes and p values are displayed in the plot captions. Mean values with 2 × SEM are presented as solid lines with tinted ribbons in a radar plot. Numbers of observations in the subsets are indicated in the X axes.

By latent class analysis (n = 422 patients) with pre-operative concentrations of sex hormones stratified by their normal range limits, three clusters of TGCT patients, termed further ‘hormonal subsets’ were identified (Supplementary Fig. S4). The *‘neutral’* subset including 54% of patients, exhibited largely normal levels of T, E2, FSH, LH and PRL. In the *‘testicle’* subset (22% or patients), roughly 70% of individuals had E2 levels above the normal range and one-third displayed supra-physiological T concentrations. This gonadal axis hyperactivity was accompanied by a suppression of the pituitary axis manifested by supra-physiological FSH and LH in nearly all testicle subset patients. The key feature of the *‘pituitary’* subset (24% of patients) was hyperactivity of the pituitary sex hormones LH and, in particular, FSH. This was paralleled by normal or lowered T and E2 levels. Differences in PRL between the hormonal subsets were significant but weak and elevated PRL were observed most frequently in the testicle subset. Differences in absolute hormone concentrations between the hormonal subsets were substantial. The elevated T and E2 concentrations in the testicle subset were paralleled by profoundly lowered FSH and LH serum levels. By contrast, the excessively high production of FSH and LH in the pituitary subset was associated with significantly decreased T and E2 concentrations (Fig. 4).

### Differences in demographic, clinical and pathological cancer features between hormonal subsets

**Figure 5:**
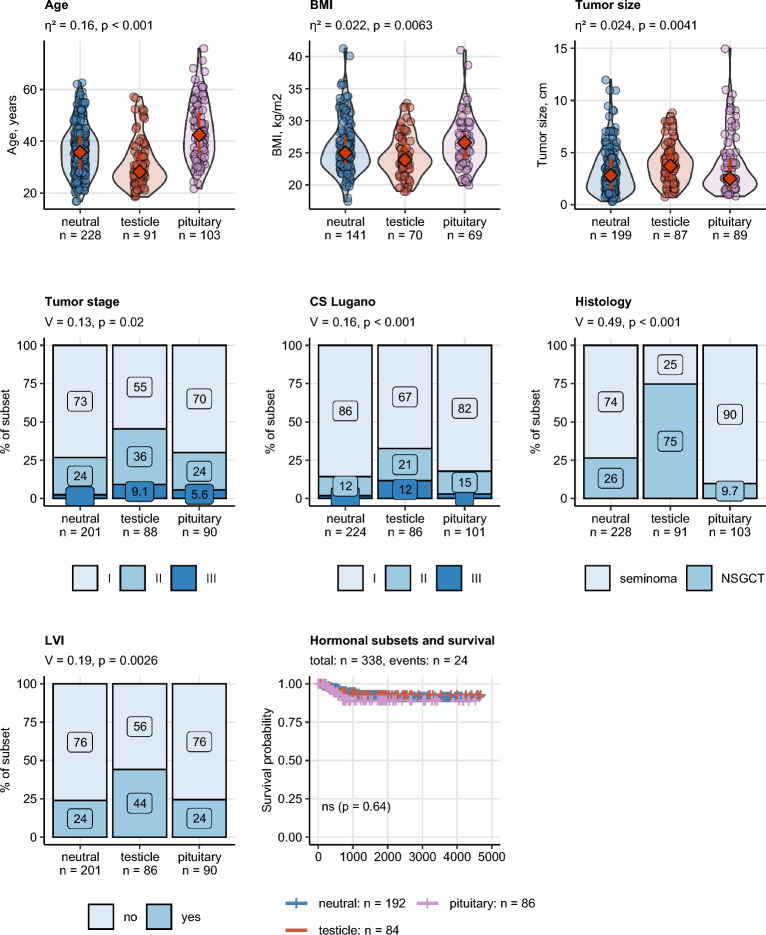
Clinico-pathological comparison of the three hormonal subsets. Differences in age, body mass index (BMI), and maximal tumor size between the hormonal subsets were assessed by Kruskal-46Wallis test with η2 effect size statistic. Differences in distribution of body mass classes, of tumor stages and Lugano classes (CS Lugano), and frequencies of lymphovascular invasion (LVI) and histology types were investigated by χ2 test with Cramer V effect size statistic. P values were corrected for multiple comparisons with the false discovery rate. Numeric variables are presented in violin plots with single observations depicted as points, and medians with interquartile ranges represented by red diamonds and whiskers. Frequencies for levels of categorical variables are shown in stack plots. Effect sizes and p values are displayed in the plot captions. Numbers of observations in the subsets are indicated in the X axes. Differences in relapse-free survival in the hormonal subsets were assessed by log-rank test. Fractions of surviving patients are displayed in a Kaplan–Meier plot. The test p value is presented in the plot. Numbers of observations and relapses are shown in the plot caption.

The newly identified hormonal subsets differed significantly in multiple demographic, clinical, pathological and therapy-related parameters (Table 4)**.** In detail, the age at surgery was the lowest in the ‘*testicle*’ subset and peaked in the ‘*pituitary*’ subset (median: 28 *vs.* 43 years; *p* < 0.001). Body mass index (BMI) was the lowest in the ‘*testicle*’ and the highest in the ‘*pituitary*’ subset (median: 24 vs 27 kg/m2; *p* = 0.0063). Accordingly, rates of overweight and obesity were the lowest in the ‘*testicle*’ subset. The ‘*testicle*’ subset included larger tumors (median: 3.7 *vs.* 2.5 cm; *p* = 0.0041) with a higher pathological (> pT1: 40% *vs.* 29.6%; *p* = 0.02) and clinical staging (> stage 1: 28% *vs.* 18%; *p* < 0.001) than the ‘*pituitary*’ subset. Interestingly, 75% of ‘*testicle*’ subset cancers were NSGCT as opposed to the ‘*pituitary*’ subset composed in 90% of seminoma. There were no significant differences in relapse-free survival between the hormonal subsets (p = 0.64) (Fig. 5). Additionally, in univariate survival Cox modeling, no significant association between absolute hormone concentrations or sex hormone strata and relapse-free survival could be discerned (not shown). Concerning postoperative management, rates of chemotherapy were peaked in the testicle subset. In turn, pituitary subset patients were more frequently administered testosterone replacement therapy during follow-up than in the remaining hormonal subsets (Supplementary Fig. S5).

**Table 4:** Demographic and clinical characteristics of hormonal subsets developed by latent class analysis in respect to preoperative sex hormone levels.

Variable^a^	Neutral	Testicle	Pituitary	Significance^b^	Effect size^b^
Age at surgery, years	36 [IQR: 29–42] Range: 17–63 Complete: n = 228	28 [IQR: 25–35] Range: 19–57 Complete: n = 91	43 [IQR: 35–52] Range: 22–76 Complete: n = 103	p < 0.001	η^2^ = 0.16
Body mass index, kg/m^2^	25 [IQR: 23–28] Range: 17–41 Complete: n = 141	24 [IQR: 22–27] Range: 19–33 Complete: n = 70	27 [IQR: 24–28] Range: 20–41 Complete: n = 69	p = 0.0063	η^2^ = 0.022
Body weight class	Normal: 50% (71) Overweight: 32% (45) Obesity: 18% (25) Complete: n = 141	Normal: 64% (45) Overweight: 30% (21) Obesity: 5.7% (4) Complete: n = 70	Normal: 38% (26) Overweight: 49% (34) Obesity: 13% (9) Complete: n = 69	p = 0.0086	V = 0.16
Tumor size, cm	2.8 [IQR: 1.5–4.5] Range: 0.3–12 Complete: n = 199	3.7 [IQR: 2.5–5] Range: 0.7–8.8 Complete: n = 87	2.5 [IQR: 1.6–4.5] Range: 0.8–15 Complete: n = 89	p = 0.0041	η^2^ = 0.024
Tumor stage	pT1: 73% (147) pT2: 24% (49) pT3: 2.5% (5) Complete: n = 201	pT1: 55% (48) pT2: 36% (32) pT3: 9.1% (8) Complete: n = 88	pT1: 70% (63) pT2: 24% (22) pT3: 5.6% (5) Complete: n = 90	p = 0.02	V = 0.13
Lugano classification	1: 86% (192) 2: 12% (28) 3: 1.8% (4) Complete: n = 224	1: 67% (58) 2: 21% (18) 3: 12% (10) Complete: n = 86	1: 82% (83) 2: 15% (15) 3: 3% (3) Complete: n = 101	p < 0.001	V = 0.16
IGCCCG risk	Good: 97% (102) Intermediate: 1.9% (2) Poor: 0.95% (1) Complete: n = 105	Good: 90% (52) Intermediate: 10% (6) Poor: 0% (0) Complete: n = 58	Good: 98% (54) Intermediate: 1.8% (1) Poor: 0% (0) Complete: n = 55	ns (p = 0.082)	V = 0.14
Rete testis infiltration	35% (70) Complete: n = 198	35% (30) Complete: n = 86	36% (32) Complete: n = 89	ns (p = 0.99)	V = 0.0077
Lymphovascular invasion	24% (48) Complete: n = 201	44% (38) Complete: n = 86	24% (22) Complete: n = 90	p = 0.0026	V = 0.19
Histology	Seminoma: 74% (168) NSGCT: 26% (60) Complete: n = 228	Seminoma: 25% (23) NSGCT: 75% (68) Complete: n = 91	Seminoma: 90% (93) NSGCT: 9.7% (10) Complete: n = 103	p < 0.001	V = 0.49
AFP, ng/mL	2.8 [IQR: 1.8–4.6] Range: 0.7–8700 Complete: n = 226	35 [IQR: 3.6–200] Range: 1–24,000 Complete: n = 91	2.5 [IQR: 1.8–3.9] Range: 1–53 Complete: n = 103	p < 0.001	η^2^ = 0.16
AFP strata	0–7 ng/mL: 87% (196) > 7 ng/mL: 13% (30) Complete: n = 226	0–7 ng/mL: 34% (31) > 7 ng/mL: 66% (60) Complete: n = 91	0–7 ng/mL: 90% (93) > 7 ng/mL: 9.7% (10) Complete: n = 103	p < 0.001	V = 0.52
HCG, IU/L	1 [IQR: 1–2] Range: 0.3–40 Complete: n = 226	77 [IQR: 23–370] range: 0.2–18,000 Complete: n = 91	1 [IQR: 1–2] Range: 0.5–96 Complete: n = 103	p < 0.001	η^2^ = 0.43
HCG strata	0–2 IU/L: 79% (178) > 2 IU/L: 21% (48) Complete: n = 226	0–2 IU/L: 5.5% (5) > 2 IU/L: 95% (86) Complete: n = 91	0–2 IU/L: 79% (81) > 2 IU/L: 21% (22) Complete: n = 103	p < 0.001	V = 0.62
LDH, U/L	190 [IQR: 160–230] Range: 3.1–920 Complete: n = 219	220 [IQR: 180–280] Range: 130–1600 Complete: n = 90	200 [IQR: 170–270] Range: 140–2900 Complete: n = 101	p = 0.0074	η^2^ = 0.021
LDH strata	0–250 U/L: 80% (176) > 250 U/L: 20% (43) Complete: n = 219	0–250 U/L: 69% (62) > 250 U/L: 31% (28) Complete: n = 90	0–250 U/L: 68% (69) > 250 U/L: 32% (32) Complete: n = 101	p = 0.031	V = 0.14
Marker status	AFP/HCG-: 70% (159) AFP/HCG + : 30% (67) Complete: n = 226	AFP/HCG-: 4.4% (4) AFP/HCG + : 96% (87) Complete: n = 91	AFP/HCG-: 70% (72) AFP/HCG + : 30% (31) Complete: n = 103	p < 0.001	V = 0.55
Retroperitoneal lymphadenectomy	6% (12) Complete: n = 200	16% (14) Complete: n = 87	4.6% (4) Complete: n = 87	p = 0.0091	V = 0.16
Chemotherapy	49% (98) Complete: n = 199	66% (57) complete: n = 87	51% (44) Complete: n = 87	p = 0.044	V = 0.13
Radiation	4.5% (9) Complete: n = 199	2.3% (2) Complete: n = 87	4.7% (4) Complete: n = 86	ns (p = 0.66)	V = 0.049
Postoperative TRT	5.1% (9) Complete: n = 177	3.8% (3) Complete: n = 80	23% (19) Complete: n = 82	p < 0.001	V = 0.28
Free testosterone, ng/mL	9.5 [IQR: 7.3–11] Range: 2.9–22 Complete: n = 62	17 [IQR: 13–28] Range: 4.4–60 Complete: n = 36	6.9 [IQR: 4.6–9.2] Range: 0.066–16 Complete: n = 34	p < 0.001	η^2^ = 0.12
SHBG, nmol/L	32 [IQR: 25–39] Range: 14–68 Complete: n = 26	20 [IQR: 15–30] Range: 6.3–160 Complete: n = 8	43 [IQR: 22–49] Range: 12–56 Complete: n = 12	ns (p = 0.22)	η^2^ = 0.0028

Numeric variables are presented as medians with interquartile ranges (IQR) and ranges. Categorical variables are presented as percentages and counts within the complete observation set.

^a^*IGCCCG* International Germ Cell Cancer Collaborative Group, *AFP* alpha fetoprotein, *LDH* lactate dehydrogenase, *SHBG* sex hormone binding globulin, *HCG* human chorionic gonadotropin, *TRT* testosterone replacement therapy.

^b^Numeric variables: Mann–Whitney test with r effect size statistic. Categorical variables: χ^2^ test with Cramer V effect size statistic. P values corrected for multiple testing with the false discovery rate method.

### Testicular tumor makers in the hormonal subsets

**Figure 6:**
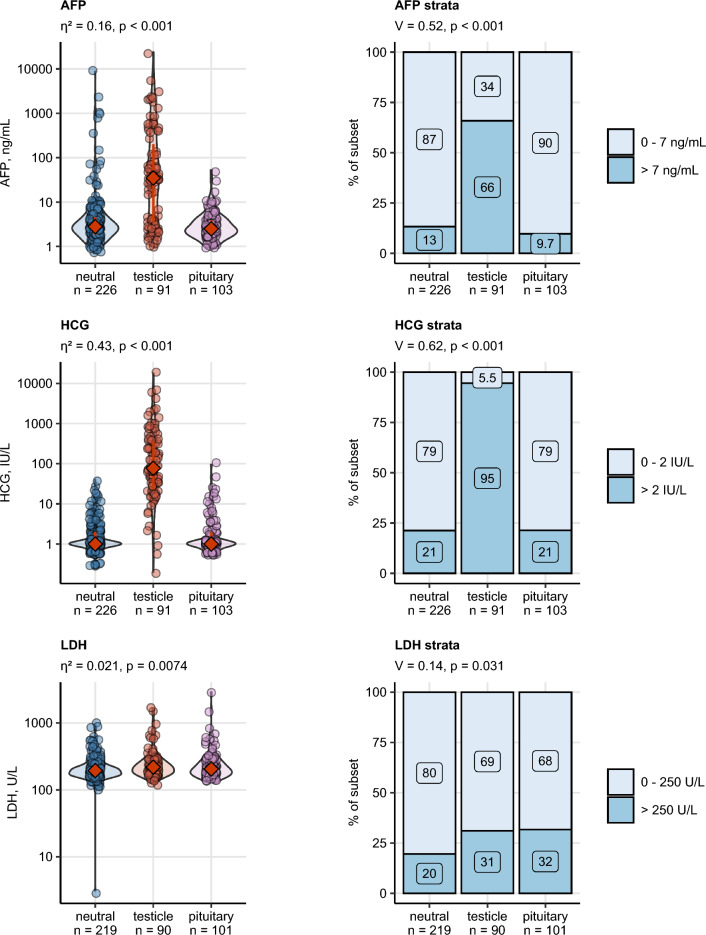
Serum tumor markers in the hormonal subsets of TGCT. Differences in blood concentrations of alpha fetoprotein (AFP) and human chorionic gonadotropin (HCG), and activity of lactate dehydrogenase (LDH) were compared between the hormonal subsets by Kruskal–Wallis test with η^2^ effect size statistic. Differences in distribution of AFP and LDH strata were assessed by χ^2^ test with Cramer V effect size statistic. P values were corrected for multiple testing with the false discovery rate method. Numeric variables are presented in violin plots with single observations depicted as points, and medians with interquartile ranges represented by red diamonds and whiskers. Frequencies for levels of categorical variables are shown in stack plots. Effect sizes and p values are displayed in the plot captions. Numbers of observations in the subsets are indicated in the X axes.

HCG differed between the hormonal subsets with the largest effect size. Nearly all patients in the ‘*testicle*’ subset showed increased HCG levels as compared with approximately 20% in the remaining subsets. Similarly, two-thirds of ‘*testicle*’ subset patients exhibited elevated AFP concentrations which was more frequent than in the ‘*neutra*l’ (13%) or ‘*pituitary*’ subset (9.7%). Differences in LDH were significant but weak with the highest activity observed in ‘*testicle*’ subset patients and the lowest activity in the ‘neutral’ subset (Fig. 6).

In line with the highly elevated HCG and AFP in the ‘testicle’ subset, nearly 96% such patients were found positive for AFP or HCG (termed further AFP/HCG + as opposed to AFP/HCG- describing patients negative for both AFP and HCG, Table 4). By contrast the AFP or HCG positivity rates in the ‘neutral’ and ‘pituitary’ subsets were approximately 30%. Herein, we characterized differences between AFP/HCG + and AFP/HCG- patients in each the ‘neutral’ and the ‘pituitary’ subset. In both the ‘neutral’ and ‘pituitary’ subset, AFP/HCG + patients had significantly larger tumors and elevated LDH as compared with the AFP/HCG- patients, suggestive for a generally higher tumor burden. Accordingly, tumor staging and Lugano classification was less favorable for the AFP/HCG + than AFP/HCG- cancers in both hormonal subsets. Both in the ‘neutral’ and ‘pituitary’ subset, AFP/HCG + tumors were more frequently classified as NSGCT as compared with AFP/HCG- cancers, which were predominantly seminoma. Interestingly, in 57% AFP/HCG + malignancies in the pituitary subset LVI was observed, which was significantly more than in AFP/HCG- patients of the same subset. No differences in LVI could be observed in the ‘neutral’ subset (Supplementary Fig. S6).

### Multi-parameter signature of the hormonal clusters of TGCT

As identified by conditional Random Forest modeling with non-endocrine explanatory factors, the most important parameter for discrimination between the hormonal subsets (neutral: n = 157, testicle: n = 72, pituitary: n = 72) was HCG, followed by age at surgery, NSGCT histology, testosterone replacement therapy, LDH, AFP, LVI and percentage of seminoma histology (Supplementary Fig. S7A). It needs to be emphasised that the Random Forest model displayed only a moderate discriminating performance (Cohen’s κ, data: 0.67, cross-validation: 0.5). In particular, whereas the ‘*neutral*’ and ‘*testicle*’ subsets could be identified with high accuracy, a large fraction of ‘pituitary’ subset patients were erroneously assigned to the ‘neutral’ subset (Supplementary Fig. S7B). This lacking sensitivity likely reflects an overall similarity of the `pituitary` and `neutral` subsets in terms of demographic, clinical and pathological features. In fact, high age at surgery and body mass index, small tumor size, the highest frequency of seminoma and the relatively most frequent testosterone replacement therapy were found to be the most specific hallmarks of the ‘pituitary’ subset (Table 4).

### Expression of sex hormone-related genes in the TCGA cohort cancers

Genes coding for the key gonadotropin-releasing hormones (*GNRH1*, *GNRH2*), pituitary gonadotropins (*PRL*, *CGA*, *LHB* and *FSH*) as well as genes involved in gonadal testosterone (*CYP11A1*, *CYP17A1*, *HSD17B3*, *HSD3B1*, *HSD3B2*) and estradiol synthesis (*CYP19A1*, *HSD17B1*), and steroid hormone transport (*SHBG*) were investigated in the TCGA cohort (Supplementary Table S2).

**Figure 7:**
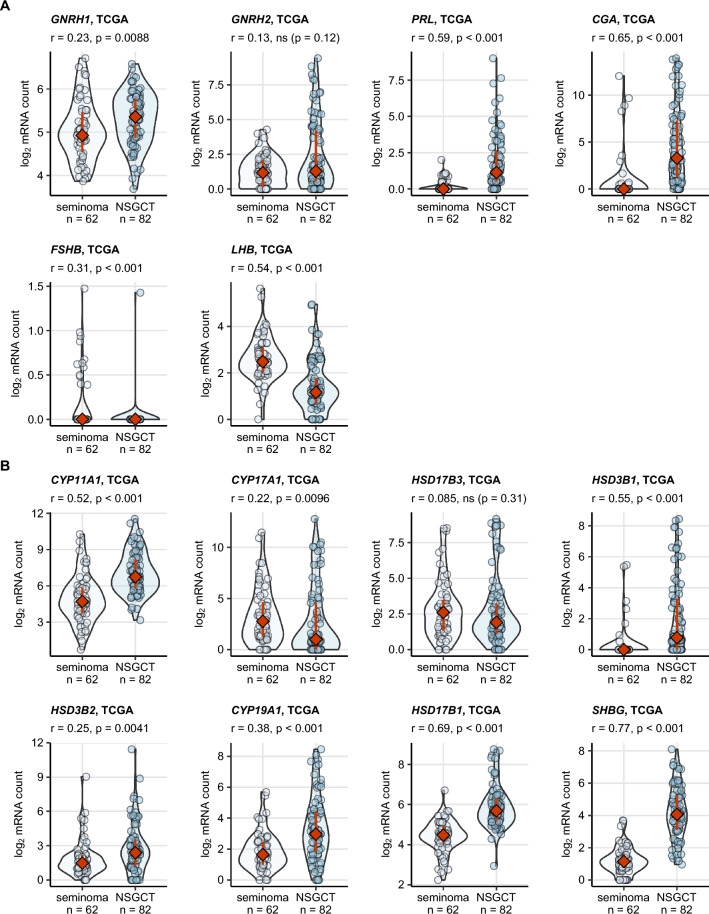
Expression of sex hormone-related genes in seminoma and NSGCT samples from the TCGA cohort. Expression of sex hormone-related genes was presented as log_2_-transformed transcript counts. (**A**) key gonadotropin-releasing hormones (*GNRH1*, *GNRH2*), pituitary gonadotropins (*PRL*, *CGA*, *LHB* and *FSHB*) as well as (**B**) genes involved in gonadal testosterone (*CYP11A1*, *CYP17A1*, *HSD17B3*, *HSD3B1*, *HSD3B2*) and estradiol synthesis (*CYP19A1*, *HSD17B1*), and steroid hormone transport (*SHBG*). Differences in gene expression between the histology types were assessed by Mann–Whitney test with r effect size statistic. P values were corrected for multiple testing with the false discovery rate method. Expression values are presented in violin plots with single samples depicted as points and red diamonds with whiskers representing medians with interquartile ranges. Effect sizes and p values are shown in the plot captions. Numbers of complete observations are indicated in the X axes.

Expression of the pituitary hormone genes was generally low, yet clearly detectable in a fraction of cancer samples. The lowest mRNA copy numbers were observed for *FSHB*. Transcripts of the investigated steroid hormone-related genes were detected in almost all cancers (Supplementary Fig. S8). Among pituitary hormone genes, *GNRH1* (weak effect), *PRL* and *CGA* (strong effect) were expressed at significantly higher levels in NSGCT than seminoma samples. Expression of *FSHB* was basically restricted to a small fraction of seminomas and *LHB* was strongly upregulated in seminoma as compared with NSGCT, in line with the differences in serum levels of our cohort. Except for the *CYP17A1* transcript which was present at moderately higher levels in seminoma and *HSD17B3* expressed at comparable levels in both histological types, the steroid hormone-related genes were upregulated in NSGCT (Fig. 7, Supplementary Table S3), in line with the generally higher serum T and E2 in NSGCT of our retrospective TGCT collective (Figs. 2, 3).

## Discussion

Testicular tumors are the most common cancer entity in young men^1^. Hormonal changes at diagnosis of TGCT have only been addressed by few publications with a limited number of patients^6–9,30^. Herein we present a comprehensive analysis of the pre-orchiectomy hormonal homeostasis in the largest collective of TGCT patients.

In this multicentric retrospective study of large number of patients with TGCT we defined by latent class analysis three hormonal subsets: first, the *‘neutral’* subset including 54% of patients, characterized by largely normal sex hormone homeostasis; second, the *‘testicle’* subset encompassing 22% of the population with clear signs of hypergonadism and suppression of the pituitary axis, and third, the *‘pituitary’* subset (24% of participants) with hyperactivity of the pituitary axis. However, in the pituitary subset, T and E2 were within the normal range or slightly lower reassembling somehow an endocrinologic compensate state. Interestingly, most cancers in the *‘testicle’* subset were NSGCTs, while in the *‘pituitary’* subset 90% of all GCTs were seminomas. Consequently, nearly 96% of the *‘testicle’* subset showed either AFP or HCG positivity in this study. Our findings are in line with previous research that had shown that high pre-orchiectomy E2, HCG levels along with decreased LH and FSH are highly specific for NSGCT^8^. A further important finding of our study is that 46% of the patients revealed a disturbed hormone homeostasis prior to surgery.

Consistent with previous reports^3^, about two-thirds of the cohort were diagnosed with pure seminomas and one third of cancers classified as NSGCT. NSGCT were characterized by a younger onset age, higher clinical stage and IGCCCG risk group, and more frequent LVI as compared with seminoma. In line with previous analyses^4,5^, we found elevated tumor markers in 43% of all patients, and significantly higher positivity rates in NSGCT. Relapse-free survival was comparable in seminoma and NSGCT in the study cohort. This results is in contrast to current understanding that clinical stage I NSGCTs have a shorter median relapse-free survival of 6 months (range, 1–75 months) than clinical stage I seminomas with a median relapse-free survival of 14 months (range, 2–84 months)^31^.

Serum levels of HCG have a paramount impact on the endocrine situation of GCT patients. While gonadal hormones were suppressed in HCG-negative cancers, HCG-positive TCGT was characterized by elevated E2 and T, and lowered LH^32^. In the “testicular” subset almost all cases revealed to be positive of AFP or HCG, and this subset consisted mainly of NSGCT with the suppression of LH and FSH. Furthermore, in our cohort, 35% of all patients were HCG-positive with a significant difference regarding the expression frequencies among seminoma and NSGCT. As previously reported, elevated HCG was found in both entities, while AFP-positivity was limited to NSGCTs^5^. We demonstrated decreased testosterone concentration in 27% of patients, while elevated levels > ULN were found in 8.6% of cases. The LH-like effect of HCG does probably trigger this effect^15^. Several patients in the “pituarity” subset revealed low normal or decreased testosterone values, whilst LH and FSH were usually elevated. Lower LH was seen in 27% of the cases, so an elevated HCG level, which was present in 35% of the cases, does not necessary results in suppression of the LH production. E2 level was out of the normal range in almost every second patient, with 16% increased and 32% decreased E2 levels. Apart from the LH-like effect of HCG, the direct hormone production of the tumours could be a possible explanation for the elevated hormone levels in some patients^13,14^. Moreover, Leydig cells, which are responsible for synthesizing testosterone, possess the two oestrogen receptors and can convert testosterone into 17beta-estradiol^33^. Previous data revealed that almost 40% of those with active disease had increased E2 levels, while in patients without active cancer only 7% demonstrated elevated E2 levels^9^. Leydig cells may produce E2 when HCG concentrations are high^7^.

If an elevated HCG is found without clinical manifestation of a TGCT, the LH level should be assessed, especially in patients with testosterone deficiency, since there can be a cross-reactivity in HCG immunoassays^5^. In addition, a higher E2 concentration may indicate a recurrence, even when other tumor markers are still within the normal range^34^.

HCG, age at surgery, NSGCT histology, LDH, AFP, LVI and percentage of seminoma histology were crucial factors for discrimination of the three hormonal subsets. As reported, the incidence peaks for seminomas at 37 years, while for NSGTC at 28 years^35^. LVI is a recognized recurrence risk factor in NSGCT, involving a risk of recurrence in 50% in the presence of LVI, while tumors without this feature have a risk of relapse in 15%^31^. Both in the ‘neutral’ and ‘pituitary’ subset, AFP/HCG + tumors were more frequently classified as NSGCT, compared to AFP/HCG-negative GCTs, which were predominantly seminoma.

The ‘*testicle*’ subset comprised of larger tumors and higher local pathological stages than the remaining subsets. A previous study did not find a significant association between clinical stage and systemic estradiol or testosterone (p = 0.104 and 0.082, respectively), however pre-orchiectomy FSH and LH were strongly correlated with larger tumor size^8^.

Following orchiectomy for TGCC, around half of patients need further treatment for occult or manifest metastatic cancer^1^, which can additionally result in decreased testosterone levels. Consequently, those patients with pre-existing Leydig cell dysfunction may be at a greater risk of testosterone deficiency^6^. Accordingly, frequent testosterone replacement therapy was one of the most specific characteristics of the *‘pituitary’* subset, with elevated LH and FSH at the time of diagnosis. Further features of this subset were higher age at surgery and increased body mass index, small tumor size and the highest frequency of seminoma. It is settled experience that with advanced age, testosterone declines^36^. Obese men have been found to have lower testosterone levels compared to men of normal weight, and the concentration of E2 was 6% higher in men who were overweight^37^. Another study did not find an association with BMI and E2 levels regardless of HCG in TGCT patients^8^. Elevated pre-orchiectomy E2 level showed a correlation with clinically significant testosterone deficiency after TGCTs treatment (p = 0.0288)^9^.

In both the ‘neutral’ and ‘pituitary’ subset, AFP/HCG + patients had larger, higher staged and, presumably, more aggressive tumors as compared with AFP/HCG- individuals^4^. Systemic inflammation, as evidenced by elevated levels of interleukin-6 and high-sensitivity C-reactive protein, has been linked to low testosterone levels in patients with metastatic cancer^38^. Bandak et al. did not find an association between disturbed Leydig cell function and clinical stage^32^. The lack of normal testicular tissue, impaired spermiogenesis and elevated E2 levels can explain the suppression of LH and FSH in larger tumors^8^.

The pathogenesis of TGCT is thought to be initiated during the fetal development of the testis^39^. According to the estrogen excess theory testicular development can be disrupted by a relatively high estrogen exposure in utero, resulting e. g. from maternal hormonal imbalance or external use of hormones. This can lead to formation of germ cell neoplasia in situ, which can be later progress upon currently unknown factors to TGCT^17^.

Our results in the TCGA cohort indicate, that a subset of TGCT expresses genes of pituitary hormones and enzymes of E2 and T biosynthesis. It remains to be investigated whether this translates to protein expression and sex hormone production by malignant cells. As such in situ synthesis of sex hormones by TGCT may further contribute to the preoperative hormonal imbalance observed by us in nearly 50% of patients. Evidence on production of sex hormones or expression of sex hormone biosynthesis genes in the TGCT tissue is scarce. Like the CYP1A1 SNPs, the SNP rs2830 of HSD17B1 was strongly correlated with NSGCT histology^16^. HSD17B1, which plays a role in conversion of estrone to E2, did not reveal a global association with TGCT risk. On the contrary, a stratified analysis of histological type showed an inverse correlation between NSGCT risk and the HSD17B1 single nucleotide polymorphism (SNP) rs2830^40^. We could detect in part strong differences between seminoma and NSGCT in expression of the sex hormone-related genes. *LHB* and *FSHB* were present at higher levels or were confined to seminoma, in line with the differences in serum hormone levels in our cohort. Almost all remaining genes including the genes of key enzymes of T and E2 biosynthesis were upregulated in NSGCT, matching with the higher T and E2 serum concentrations in NSGCT of our collective. The production of steroids in the testes depends on the Leydig cells^41^. A study indicated that the CYP1A2 variant rs762551 was inversely associated with TGCT risk^40^.

Several limitations must be mentioned. Multiple potentially interesting variables such as SHBG or free T levels, were affected by high missingness precluding their use in LCA or survival modeling. Of note, free T was shown to be a more reliable marker of T deficiency than the total hormone levels. LCA modeling outcome is affected by definition of qualitative explanatory variables, in our case, the hormone level strata. In particular, the use of age-independent cutoffs for the definition of testosterone insufficiency is controversial^42^. Due to the retrospective study design, complete data on cryptorchidism and testicular volume was not available. Regarding previous publications, cryptorchidism is a well-known risk factor for testicular cancer, mainly for seminomas and can result in endocrine and/or exocrine dysfunction of the testes^43^. Furthermore, testicular volume is reported to positively correlate with testosterone and negatively correlate with LH and FSH, thus testicular volume could have an impact on the hormonal axis as well^44,45^.

## Conclusions

Nearly 50% of TGCT patients are at an increased risk of sex hormone imbalances before orchiectomy. We identified three subsets of TGCT characterized by normal sex hormone homeostasis, hypergonadism with consecutive suppression of the pituitary axis, or hyperactivity of the pituitary axis, respectively. In our large TCGT collective, no differences in relapse risk attributed to the cancer histology type, pre-operative sex hormone levels or hormonal subset assignment could be observed. We demonstrated measurable expression of sex hormone biosynthesis genes in a subset of TGCT. Whether sex hormone production in the malignant tissue participates in systemic hormonal imbalance in testicular cancer, remains to be further investigated.

### Supplementary Information

Supplementary Information.

## Data Availability

The retrospective cohort data is available upon request. The TCGA cohort data is available via cBioPortal (https://www.cbioportal.org/). The entire R analysis pipeline is available as a GitHub repository (https://github.com/PiotrTymoszuk/TesCa_hormones).

## Abbreviations

AFP: Alpha-fetoprotein
BIC: Bayesian information criterion
C: Harrell’s concordance index
CT: Computed tomography
EAU: European association of urology
E2: Estradiol
FSH: Follicle-stimulating hormone
GCNIS: Germ cell neoplasia in situ
GCT: Germ cell tumors
hCG: Human chorionic gonadotropin
HR: Hazard ratio
IBS: Integrated brier score
LCA: Latent class analysis
LDH: Lactate dehydrogenase
LH: Luteinizing hormone
MCA: Multi-dimensional correspondence analysis
NSGCT: Non-seminomatous germ cell tumors
PCA: Principal component analysis
PRL: Prolactine
RFS: Relapse-free survival
ROC: Receiver-operating characteristic
SHBG: Sex-hormone binding globuline
SNP: Single nucleotide polymorphism
T: Testosterone
TRT: Testosterone replacement therapy
ULN: Upper limit of norm
