# Supplementary material for: Subsets of preoperative sex hormones in testicular germ cell cancer: a retrospective multicenter study
Source: Sci Rep. 2023 Sep 5;13:14604. doi: 10.1038/s41598-023-41915-7 (PMC10480169; doi:10.1038/s41598-023-41915-7)
Supplement: Supplementary file 1 — Supplementary Information. [file 41598_2023_41915_MOESM1_ESM.pdf]

Supplementary Methods

Data sources

The retrospective cohort study dataset was provided as an Excel sheet. Data import in R was accomplished with the `read_xslx()` R function (package *readxl*) (1). The dataset was formatted with an in-house developed script.

Predominantly embryonic cancers, predominantly teratoma, predominantly yolk sac cancers and predominantly chorionic cancers were defined by the cutoff of ≥ 75% of the respective histology in pathological examination. Tumor stages and Lugano clinical stages were expressed in simplified form as 'I', 'II', 'III'. Laboratory parameter values beyond the detection range were set to their respective lower and upper detection limit.

Laboratory parameters were stratified as follows:

- lactate dehydrogenase (LDH): normal: 0 - 250 U/L, elevated: > 250 U/L
- alpha fetoprotein: normal: 0 - 7 ng/mL, elevated: > 7 ng/mL
- human chorionic gonadotropin (HCG): normal: 0 - 2 IU/L, elevated: > 2 IU/L
- total testosterone (T total): low: 0 - 3.5 ng/mL, normal: 3.5 - 9 ng/mL, elevated: > 9 ng/mL
- estradiol (E2): low: 0 - 20 pg/mL, normal: 20 - 55 pg/mL, elevated: > 55 pg/mL
- follicle stimulating hormone (FSH): low: 0 - 1 mU/mL, normal: 1 - 10 mU/mL, elevated: > 10 mU/mL
- luteinizing hormone (LH): low: 0 - 1.7 mU/mL, normal: 1.7 - 8.6 mU/mL, elevated: > 8.6 mU/mL
- prolactin (PRL): normal: 0 - 480 µU/mL, elevated: > 480 µU/mL

Tumor marker positivity (AFP/HCG+) was defined as at least one of AFP or HCG being elevated according to the cutoffs presented above.

In the current analysis, n = 518 testis cancer patients of the retrospective cohort with ≤ 50% missing variables were included (**Supplementary Figure S1**).

The testicle cancer dataset (clinical information, expression) of the TCGA pan-cancer project (2) with n = 149 specimens was fetched from cBioPortal. The R import and formatting was accomplished with an in-house developed script.

Software

Data analysis was done with R version 4.2.3.

Tabular data was handled with the *tidyverse* package bundle (3), *rlang* (4) and *trafo*. Text data manipulation and search was accomplished with *stringi* (5).

Descriptive statistics were computed with *DescTools* (6), *rstatix* (7) and *ExDA*. Statistical hypothesis testing was done for numeric and categorical variables with the packages *rstatix* (7) and *ExDA*, and with the *survival* (8) and *survminer* (9) packages for survival data.

Principal component analysis (PCA) was performed with the development package *clustTools* employing the pcaPP algorithm by Croux et al. (10,11). Multi-dimensional correspondence analysis (MCA) was done with the *MASS* package (12). Hormonal subsets of testicle cancers in the retrospective cohort were defined by maximum-likelihood latent class analysis (LCA) done with *poLCA* (13,14).

Multi-parameter classifiers of hormonal subsets of testicular cancer were established for the retrospective cohort with the conditional Random Forest algorithm (15,16) provided by the package *party* (17). For cross-validation, computation of fit statistics and quality control of the Random Forest models, the packages *caret* (18) and the development package *caretExtra*.

Analysis results were visualized with *ggplot2* (19) (bar, scatter and bubble plots, heat maps), *ExDA* (violin, box, stack and radar plots), *caretExtra*, *survminer* (9) (Kaplan-Meier plots), *clustTools* (PCA loadings and MCA column factors). Report figures were generated with *cowplot* (20). Tables were created with *flextable* (21).

Supplementary Material and parts of the manuscript were written in the *rmarkdown* environment (22) with the package *bookdown* (23). Management of figures, tables, links and references in the rmarkdown document was accomplished with *figur*. The report was rendered as a html document with *knitr* (24) and *rmdformats* (25).

Statistical significance and multiple testing adjustment

If not indicated otherwise, statistical testing p values were corrected for multiple testing with the false discovery rate method (FDR) (26) within each analysis task. Effects with FDR-corrected $p < 0.05$ are considered statistically significant. Effect sizes for categorical variables were defined with the Cramer V effect size statistic with the following cutoffs: ≤ 0.3: weak, 0.3 - 0.6: moderate, > 0.6: strong. Effect sizes for numeric variables analyzed with Mann-Whitney test were assessed by r effect size statistic with the following cutoffs: ≤ 0.3: small, 0.3 - 0.5: moderate; > 0.5: strong. Effect sizes for numeric variables analyzed with Kruskal-Wallis test were investigated by η^2 effect size metrics with the following cutoffs: ≤ 0.13: weak, 0.13 - 0.26: moderate, > 0.26: strong. Accuracy was assessed by

Cohen's κ inter-rater reliability measure with the following effect size cutoffs: ≤ 0.4: weak, 0.4 - 0.6: moderate, 0.6 - 0.8: good, > 0.8: very good.

Explorative data analysis

Numeric variables are presented in the text and tables as medians with interquartile ranges (IQR), ranges and numbers of complete observations. Qualitative variables are presented as percentages and counts of the categories within the complete observation set. Descriptive characteristic of the retrospective and TCGA cohorts was generated with the `explore(what = 'table', pub_styled = TRUE)` function (package *ExDA*) and is presented in **Table 1**, **Table 3** and **Supplementary Table S2**.

Normality of numeric variables was assessed with Shapiro-Wilk test (function `explore()`, package *ExDA* and quantile-quantile plots. Since multiple numeric variables were not-normally distributed, non-parametric statistical hypothesis tests were used.

Comparison of seminoma and non-seminomatous germ cell tumors in the retrospective cohort

Demographic, clinical, pathological, therapy-related and endocrine parameters were compared between the histology types with Mann-Whitney test and r effect size statistic (numeric variables) or χ^2 test with Cramer V effect size statistic (function `compare_variables()`, package *ExDA*). The comparison results are presented in **Table 2**, **Figures 1 - 3** and **Supplementary Figure S2**. Differences in survival between the histology types were analyzed with Peto-Peto test (function `surv_pvalue()`, package *survminer*, **Figure 1**).

Co-regulation of pre-surgery sex hormones in the retrospective cohort

Co-regulation of pre-surgery sex hormones treated as normalized, median-centered absolute concentrations were investigated by 4-dimensional PCA (10,11) (function `reduce_data(kdim = 4, red_fun = 'pca')`, package *clustTools*). The first four components of the PCA explained >90% of the entire hormone dataset variance as investigated by visual analysis of the scree plot (not shown). PCA loadings for the first two major principal components were visualized with the `plot(type = 'loadings')` method (package *clustTools*).

Trends or overlaps in strata of sex hormone levels (concentrations stratified by limits of reference ranges) were explored by two-dimensional MCA performed with the `mca()` function from the *MASS* package (12). Column factors were visualized with a custom script.

A total of n = 422 observations were available for these analyses. The results of component and correspondence analysis are presented in **Supplementary Figure S3**.

Definition of hormonal subsets with latent class analysis in the retrospective cohort

To define subsets of testicular cancers based on clinical strata of pre-surgery sex hormone levels (categorical variables, absolute concentrations stratified by limits of their reference ranges, available observations: n = 422) termed further 'hormonal subsets', LCA was employed (5000 iterations, 5 algorithm runs, function `poLCA(maxiter = 5000, nrep = 5)`, package *poLCA*) (13,14).

The optimal number of hormonal subsets was determined by comparing values of Bayesian Information Criterion (BIC) for LCA models with varying number of classes. The LCA solution with k = 3 classes/hormonal subsets displayed the minimal BIC value suggestive of the optimal model fit. Convergence of this model was achieved in n = 1393 iterations out of 5000 iterations in total (**Figure 4A**). Conditional probabilities for the hormonal subset assignment estimates for the hormone concentration strata by the final LCA model are presented in **Supplementary Figure S4**.

Differences in distribution of sex hormone strata between the hormone subsets were assessed by χ^2 test with Cramer V effect size statistic. Differences in blood hormone concentrations expressed as numeric variables between the hormone subsets were investigated by Kruskal-Wallis test with η^2 effect size statistic (both comparison types done with `compare_variables()`, package *ExDA*). Differences in sex hormone levels between the hormonal subsets are shown in **Figure 4** and **Supplementary Table 1**.

Demographic and clinical characteristic of the hormonal subsets of the retrospective cohort

Demographic, clinical, pathological and therapy-related parameters were compared between the hormonal subsets with Kruskal-Wallis test and η^2 effect size statistic (numeric variables) or χ^2 test with Cramer V effect size statistic (categorical variables; function `compare_variables()`, package *ExDA*). The comparison results are presented in **Table 4**, **Figures 5 - 6** and **Supplementary Figure S@ref(fig:fig-subsets-therapy**. Additionally, neutral and pituitary hormonal subset patients were split according to their AFP and HCG status (negative for both AFP/HCG versus positive for AFP or HCG) and differences between the status strata compared with Mann-Whitney test with r effect size statistic or χ^2 test with Cramer V effect size statistic, as appropriate. Significant differences identified in this analysis are presented in **Supplementary Figure S6**.

Multi-paramater discrimination between the hormonal subsets of the retrospective cohort

A multi-parameter model allowing for discrimination between the hormone subsets with solely non-endocrine explanatory variables (available observations: n = 305, **Supplementary Figure S7A**) was established with the conditional Random Forest algorithm (15–17) using the `train(method = 'cforest')` wrapper function from the package *caret* (18). The optimal `mtry` parameter value obtained by cross-validation-based tuning with Cohen's κ as the tuning metric was 18. The remaining model parameters such as split rule (set to the maximum test statistic), splitting criterion (set to 1.28) and number of random trees (1000) were specified in the control objects returned by the `cforest_classical()` function from the package *party*.

Model performance in the training dataset and 10-fold cross-validation was assessed by the accuracy and Cohen's κ statistics computed with the `summary()` method (package *caretExtra*) and are presented in **Supplementary Figure S7B**. Confusion matrices of the predicted and actual hormone subset assignment were visualized with the `plot(type = 'confusion')` method provided by the *caretExtra* package. Permutation importance for explanatory variables was computed as described for the histology types and is presented in **Supplementary Figure S7A**.

Expression of sex hormone-related genes in the TCGA cohort

Expression of 14 sex hormone-related genes in the tumor tissue of the TCGA cohort specimens was investigated in the current report (*GNRH1, GNRH2, PRL, CGA, FSHB, LHB, CYP11A1, CYP17A1, HSD17B3, HSD3B1, HSD3B2, CYP19A1, HSD17B1, SHBG*). Differences in gene expression between seminoma and NSGCT samples were investigated by Mann-Whitney U test with r effect size statistic (function `compare_variables()`, package *ExDA*). Trends in regulation of the gene expression were revealed by 8-dimensional PCA (function `reduce_data(red_fun = 'pca', kdim = 8)`, package *clustTools*).

Supplementary tables

Supplementary Table S1: Differences in preoperative absolute serum concentrations of sex hormones between the hormonal subsets of the retrospective cohort. Concentrations are presented as medians with interquartile ranges (IQR) and ranges.

Variable[a]	Neutral	Testicle	Pituitary	Significance[b]	Effect size[b]
Participants, n	228	91	103		
E2, pg/mL	25 [IQR: 16 - 33]<br>range: 0.5 - 69	79 [IQR: 52 - 130]<br>range: 8 - 200	24 [IQR: 17 - 30]<br>range: 5 - 60	$p < 0.001$	$\eta^2 = 0.35$
Total testosterone, ng/mL	4.3 [IQR: 3.2 - 5.4]<br>range: 1 - 11	8.1 [IQR: 5.8 - 9.9]<br>range: 1.7 - 15	3.8 [IQR: 2.8 - 4.6]<br>range: 1.4 - 10	$p < 0.001$	$\eta^2 = 0.3$
FSH, mU/mL	5.1 [IQR: 3.6 - 7.4]<br>range: 1.2 - 10	0 [IQR: 0 - 0.2]<br>range: 0 - 2.7	16 [IQR: 12 - 24]<br>range: 10 - 100	$p < 0.001$	$\eta^2 = 0.82$
LH, mU/mL	3.3 [IQR: 2.3 - 4.4]<br>range: 0.4 - 20	0 [IQR: 0 - 0.3]<br>range: 0 - 11	6.9 [IQR: 4.9 - 10]<br>range: 2.3 - 41	$p < 0.001$	$\eta^2 = 0.6$
PRL, µU/mL	160 [IQR: 97 - 210]<br>range: 0 - 4400	250 [IQR: 160 - 350]<br>range: 0 - 650	170 [IQR: 100 - 270]<br>range: 0 - 1300	$p < 0.001$	$\eta^2 = 0.092$

[a]T: total testosterone; E2: estradiol; FSH: follicle-stimulating hormone; LH: luteinizing hormone; PRL: prolactin,

[b]Kruskal-Wallis test with η^2 size statistic. P values corrected for multiple testing with the false discovery rate method.

Supplementary Table S2: Characteristic of the TCGA cohort. Numeric variables are presented as medians with interquartile ranges (IQR) and ranges. Categorical variables are presented as percentages and counts within the complete observation set.

**Variable**[a]	**Statistic**
Age at surgery, years	31 [IQR: 26 - 37]<br>range: 14 - 67<br>complete: n = 133
Race/Ethnicity	Asian: 3.1% (4)<br>Black or African American: 4.7% (6)<br>White: 92% (118)<br>complete: n = 128
Tumor stage	I: 84% (102)<br>II: 9% (11)<br>III: 7.4% (9)<br>IV: 0% (0)<br>complete: n = 122
Metastasis stage	M0: 97% (114)<br>M1: 3.4% (4)<br>complete: n = 118
Node stage	N0: 79% (46)<br>N1: 17% (10)<br>N2: 3.4% (2)<br>complete: n = 58
IGCCCG risk	good: 74% (32)<br>intermediate: 21% (9)<br>poor: 4.7% (2)<br>complete: n = 43
Histology	seminoma: 43% (62)<br>NSGCT: 57% (82)<br>complete: n = 144
Marker status (AFP, HCG, LDH)	S0: 36% (43)<br>S1: 31% (37)<br>S2: 29% (34)<br>S3: 4.2% (5)<br>complete: n = 119
Radiation	16% (21)<br>complete: n = 130

[a]IGCCCG: International Germ Cell Cancer Collaborative Group.

Supplementary Table S3: Differences in expression of the hormone-related genes of interest between seminoma and NSGCT in the TCGA cohort. Numeric variables are presented as medians with interquartile ranges (IQR) and ranges. Categorical variables are presented as percentages and counts within the complete observation set.

**Variable**	**Seminoma**	**NSGCT**	**Significance**[a]	**Effect size**[a]
Samples, n	62	82		
GNRH1	4.9 [IQR: 4.5 - 5.5]<br>range: 3.9 - 6.7	5.4 [IQR: 4.9 - 5.8]<br>range: 3.7 - 6.6	p = 0.0088	r = 0.23
GNRH2	1.2 [IQR: 0.12 - 2]<br>range: 0 - 4.3	1.3 [IQR: 0.56 - 4.2]<br>range: 0 - 9.4	ns (p = 0.12)	r = 0.13
PRL	0 [IQR: 0 - 0]<br>range: 0 - 2	1.1 [IQR: 0.5 - 2.6]<br>range: 0 - 9	p < 0.001	r = 0.59
CGA	0 [IQR: 0 - 0]<br>range: 0 - 12	3.3 [IQR: 1.2 - 7.3]<br>range: 0 - 14	p < 0.001	r = 0.65
FSHB	0 [IQR: 0 - 0]<br>range: 0 - 1.5	0 [IQR: 0 - 0]<br>range: 0 - 1.4	p < 0.001	r = 0.31
LHB	2.5 [IQR: 2 - 3.1]<br>range: 0 - 5.6	1.2 [IQR: 0.69 - 1.8]<br>range: 0 - 5	p < 0.001	r = 0.54
CYP11A1	4.7 [IQR: 3.7 - 5.9]<br>range: 0.73 - 10	6.8 [IQR: 5.8 - 8.2]<br>range: 3.2 - 12	p < 0.001	r = 0.52
CYP17A1	2.8 [IQR: 1.3 - 4.6]<br>range: 0 - 11	1 [IQR: 0 - 4.5]<br>range: 0 - 13	p = 0.0096	r = 0.22
HSD17B3	2.6 [IQR: 1.3 - 3.5]<br>range: 0 - 8.5	1.9 [IQR: 1.1 - 3.2]<br>range: 0 - 9.2	ns (p = 0.31)	r = 0.085
HSD3B1	0 [IQR: 0 - 0]<br>range: 0 - 5.5	0.77 [IQR: 0 - 3.3]<br>range: 0 - 8.4	p < 0.001	r = 0.55
HSD3B2	1.5 [IQR: 1 - 2.2]<br>range: 0 - 9	2.4 [IQR: 1.2 - 3.5]<br>range: 0 - 11	p = 0.0041	r = 0.25
CYP19A1	1.6 [IQR: 0.99 - 2.5]<br>range: 0 - 5.7	3 [IQR: 1.8 - 4.5]<br>range: 0 - 8.5	p < 0.001	r = 0.38
HSD17B1	4.5 [IQR: 4.1 - 4.9]<br>range: 2.2 - 6.7	5.7 [IQR: 5.2 - 6.3]<br>range: 2.9 - 8.8	p < 0.001	r = 0.69
SHBG	1.1 [IQR: 0.73 - 1.6]<br>range: 0 - 3.7	4.1 [IQR: 3.1 - 5.2]<br>range: 0.96 - 8.1	p < 0.001	r = 0.77

Variable	Seminoma	NSGCT	Significance[a]	Effect size[a]

[a]Mann-Whitney test with r effect size statistic. P values corrected for multiple testing with the false disocvery rate method.

Supplementary figures

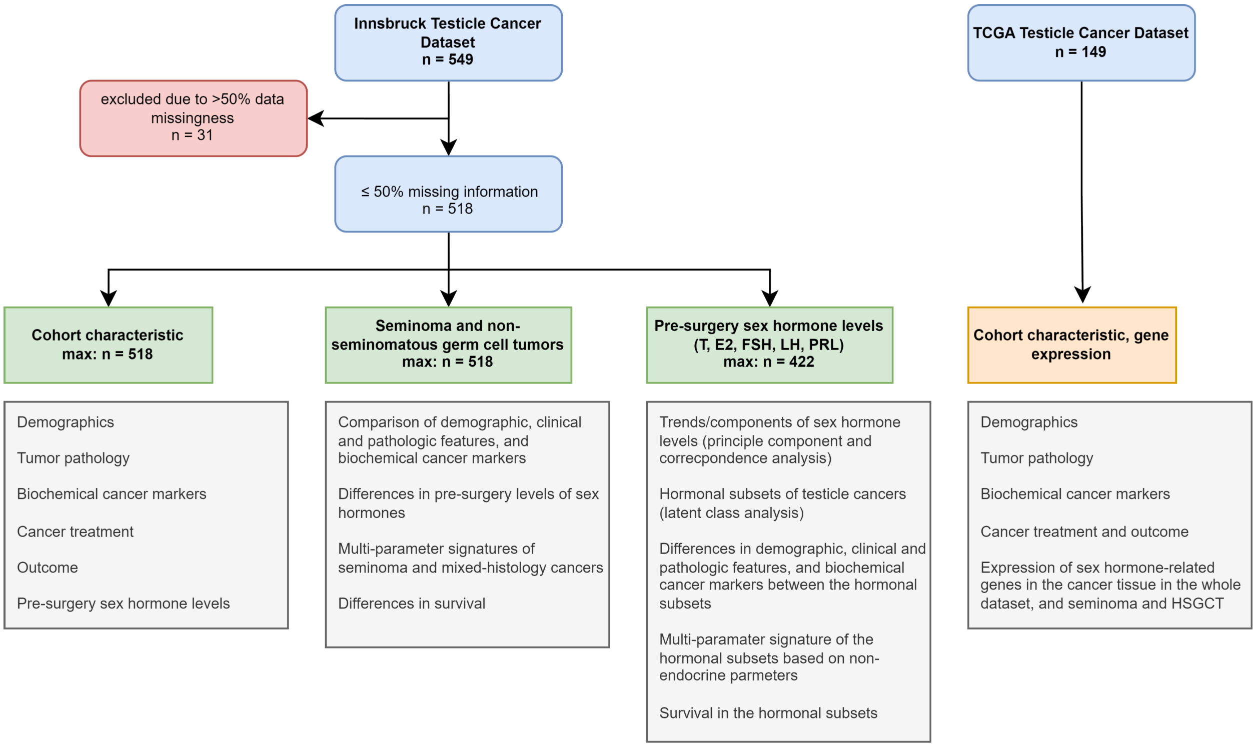

Supplementary Figure S1. Analysis inclusion scheme and data analysis strategy.

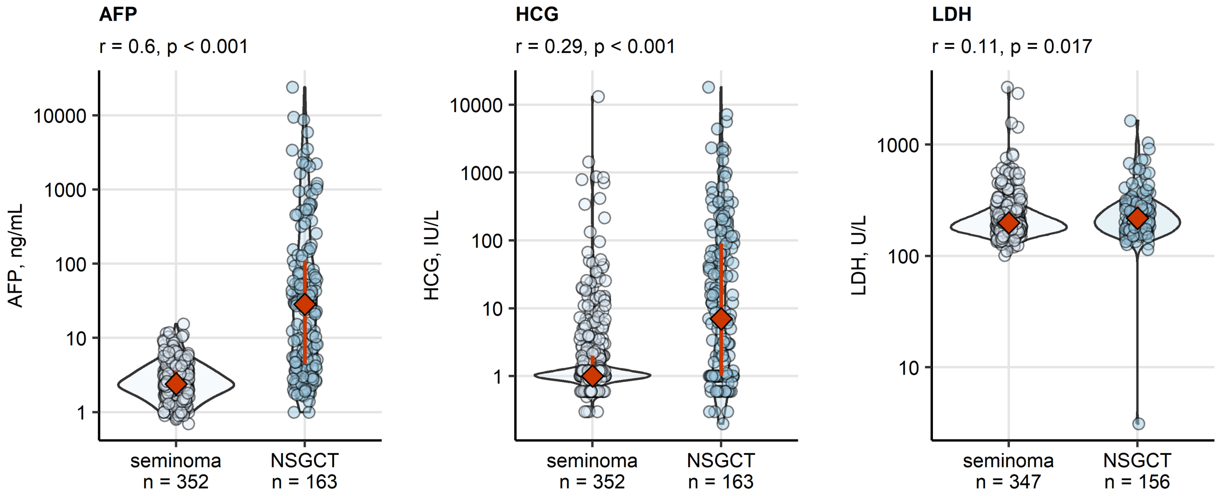

Supplementary Figure S2. Preoperative absolute serum concentrations of cancer markers in seminoma and non-seminomatous germ cell tumors.

Differences in serum concentrations of alpha fetoprotein (AFP), human chorionic gonadotropin (HCG) and activity of lactate dehydrogenase (LDH) between seminoma and non-seminomatous germ cell tumors (NSGCT) were assessed by Mann-Whitney test with r effect size statistic. P values were corrected for multiple testing with the false discovery rate method. Concentrations are presented in violin plots wit single observations depicted as points and medians with interquartile ranges represented by red diamonds and whiskers. Effect sizes and p values are displayed in the plot captions. Numbers of complete observations are indicated in the X axes.

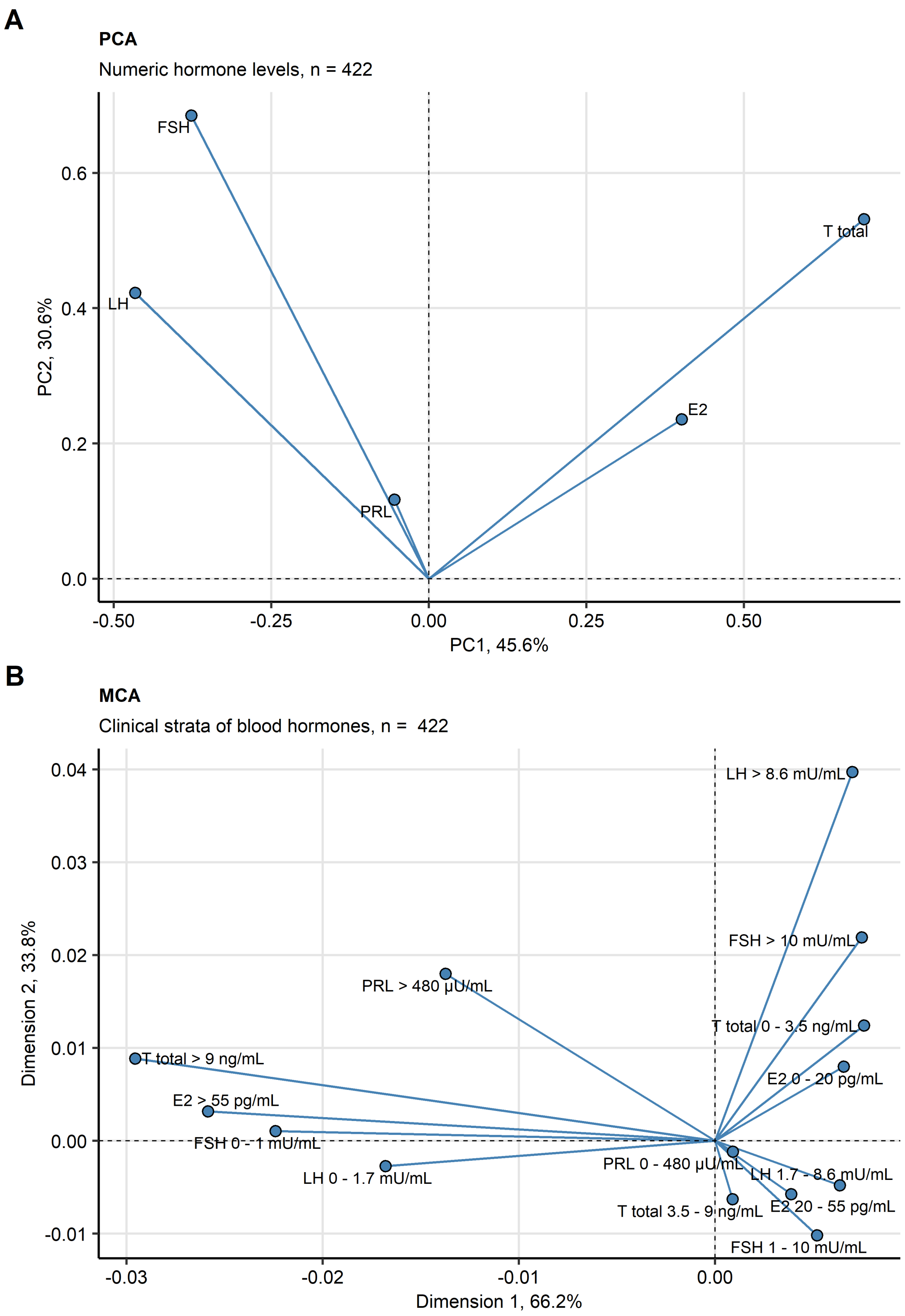

Supplementary Figure S3. Principal component and correspondence analysis of pre-surgery sex hormone levels.

Blood concentrations of sex hormones (T: testosterone, E2: estradiol, FSH: follicle stimulating hormone, LH: luteinizing hormone; PRL: prolactin) as normalized median-centered numeric variables and as clinical strata were subjected to 4-dimensional principal component analysis (PCA, A) and multidimensional correspondence analysis (MCA, B), respectively. Loadings (PCA) and column factors (MCA) for the first two dimensions are presented in scatter plots plots. Numbers of observations are indicated in the plot captions.

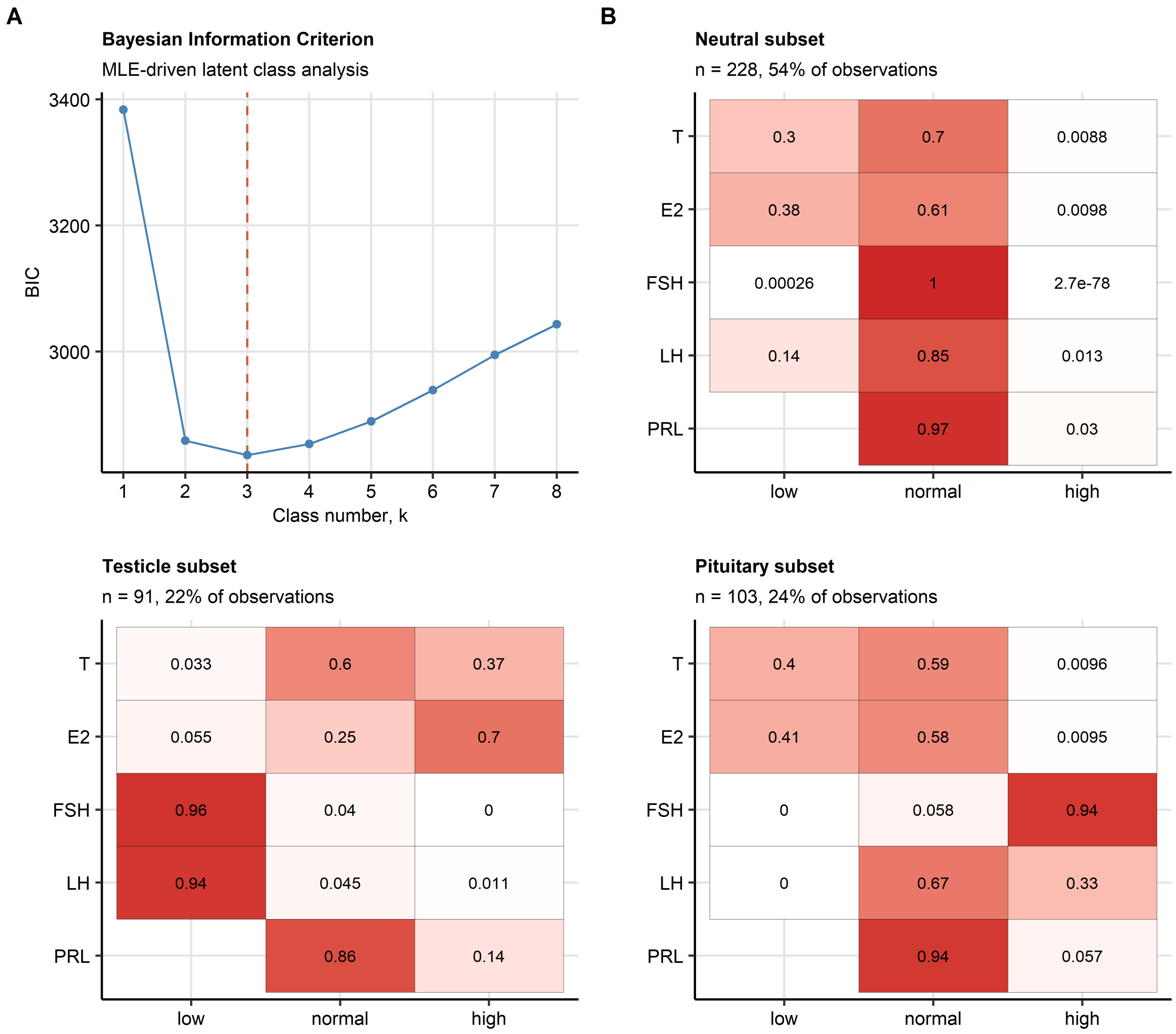

Supplementary Figure S4. Definition of the hormonal subsets with latent class analysis.

Hormonal subsets of testicle cancer were defined by latent class analysis (LCA) employing pre-surgery blood concentration of sex hormones (T: testosterone, E2: estradiol, FSH: follicle stimulating hormone, LH: luteinizing hormone, PRL: prolactin) stratified by limits of their reference ranges.

(A) Goodness of fit of the LCA models for various subset numbers was measured by Bayesian Information Criterion (BIC). Note: the goodness-of-fit improves with decreasing BIC. BIC values for the investigated class numbers are presented in a line plot. The vertical dashed line indicates the subset number chosen for the final model.

(B) Hormonal subset conditional response probabilities estimated by the final LCA model presented as heat maps. Numbers of observations in the subsets and percentage within the hormone dataset are indicated in the plot captions.

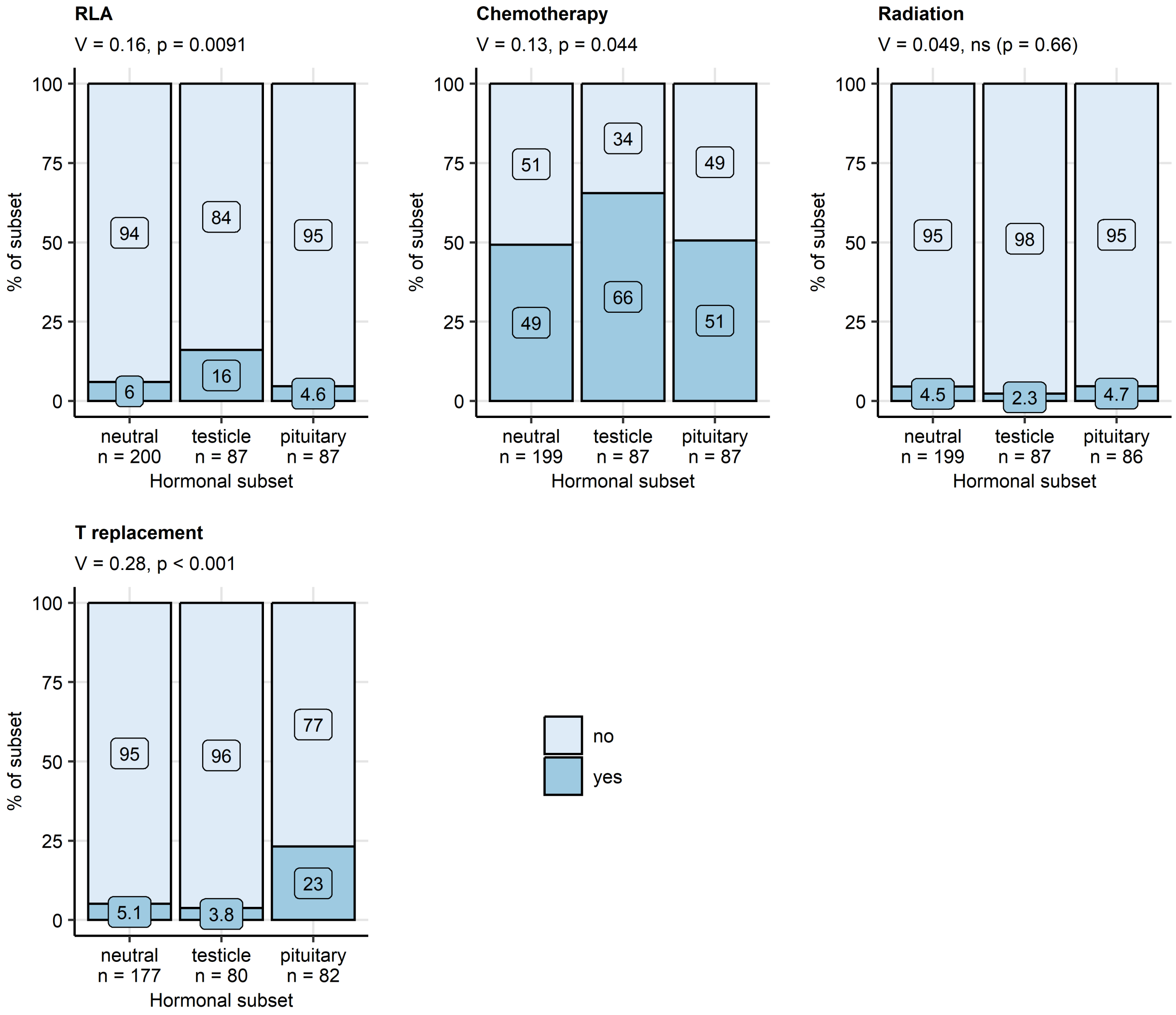

Supplementary Figure S5. Therapy in the hormonal subsets of the retrospective cohort.

Rates of retroperitoneal lymphadenectomy (RLA), chemo- and radiotherapy in the hormonal subsets were compared by χ^2 test with Cramer V effect size statistic. P values were corrected for multiple testing with the false discovery rate method. Therapy frequencies in the subsets are presented in stack plots. Effect sizes and p values are displayed in the plot captions. Numbers of observations in the subsets are indicated in the X axes.

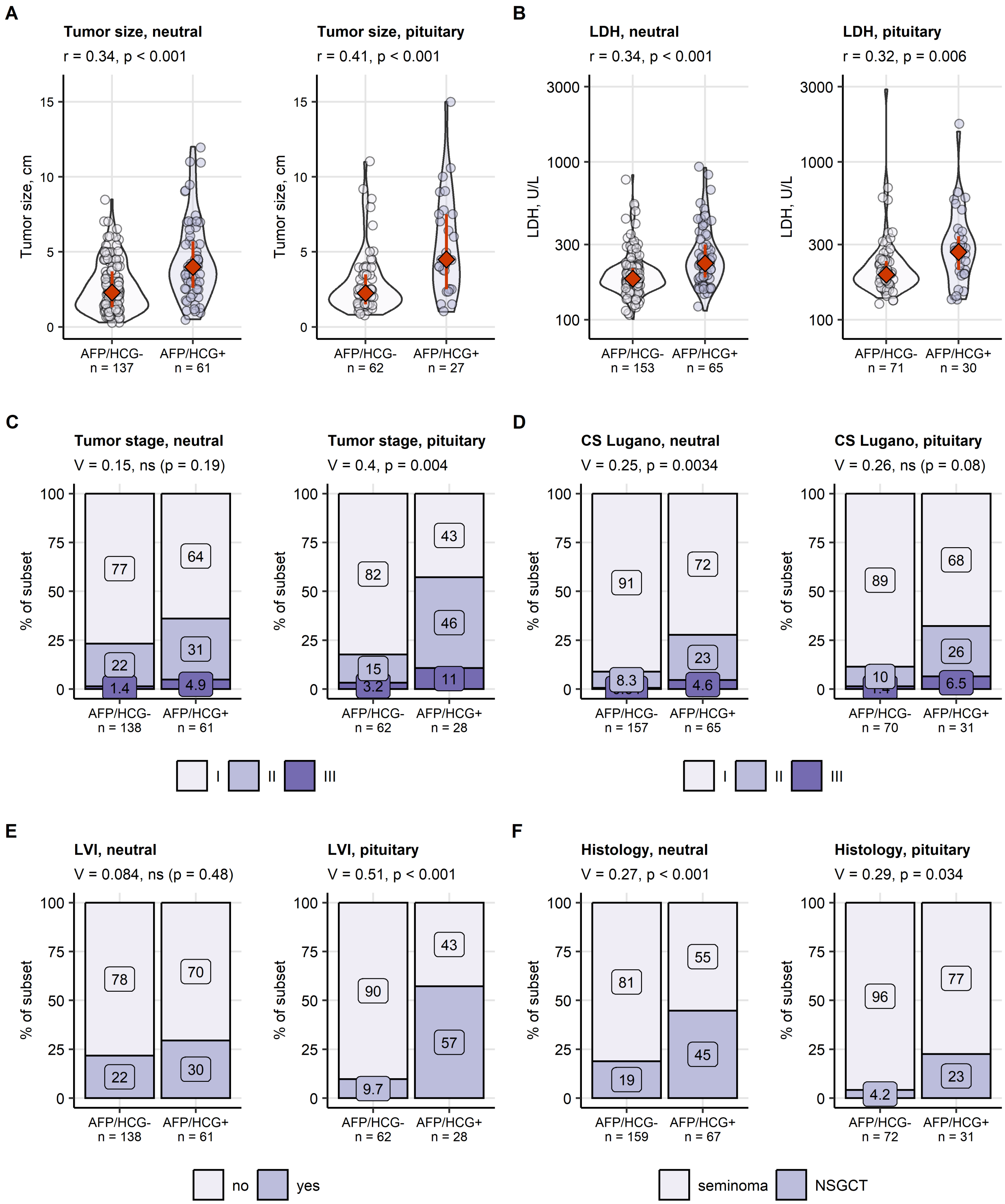
A
Tumor size, neutral
r = 0.34, p < 0.001
Tumor size, pituitary
r = 0.41, p < 0.001
Tumor size, cm
AFP/HCG-
n = 137
AFP/HCG+
n = 61
AFP/HCG-
n = 62
AFP/HCG+
n = 27
B
LDH, neutral
r = 0.34, p < 0.001
LDH, pituitary
r = 0.32, p = 0.006
LDH, U/L
AFP/HCG-
n = 153
AFP/HCG+
n = 65
AFP/HCG-
n = 71
AFP/HCG+
n = 30
C
Tumor stage, neutral
V = 0.15, ns (p = 0.19)
Tumor stage, pituitary
V = 0.4, p = 0.004
% of subset
AFP/HCG-
n = 138
AFP/HCG+
n = 61
AFP/HCG-
n = 62
AFP/HCG+
n = 28
I
II
III
D
CS Lugano, neutral
V = 0.25, p = 0.0034
CS Lugano, pituitary
V = 0.26, ns (p = 0.08)
AFP/HCG-
n = 157
AFP/HCG+
n = 65
AFP/HCG-
n = 70
AFP/HCG+
n = 31
E
LVI, neutral
V = 0.084, ns (p = 0.48)
LVI, pituitary
V = 0.51, p < 0.001
AFP/HCG-
n = 138
AFP/HCG+
n = 61
AFP/HCG-
n = 62
AFP/HCG+
n = 28
no
yes
F
Histology, neutral
V = 0.27, p < 0.001
Histology, pituitary
V = 0.29, p = 0.034
AFP/HCG-
n = 159
AFP/HCG+
n = 67
AFP/HCG-
n = 72
AFP/HCG+
n = 31
seminoma
NSGCT

Supplementary Figure S6. Differences in tumor pathology between neutral and pituitary hormonal subset participants split by the alpha fetoprotein and human chorionic gonadotropin status.

The retrospective cohort patients were stratified according to positivity of alpha fetoprotein (AFP, cutoff: 7 ng/mL) and human chorionic gonadotropin (HCG, cutoff: 2 IU/L): the AFP/HCG- patients were negative for both markers and AFP/HCG+ patients were positive for at least one of the markers. Subsequently, differences in study variables between AFP/HCG- and AFP/HCG+ patients assigned to the neutral and pituitary hormone subset were investigated. Differences in maximal tumor size (A) and lactate dehydrogenase activity (LDH, B) were assessed by Mann-Whitney test with r effect size statistic. Differences in tumor stages (C), Lugano classes (D), rates of lymphovascular invasion (LVI, E) and distribution of histological types (NSGCT: non-seminomatous germ cell tumors, F) were assessed by χ^2 *test with Cramer V effect size statistic. P values were corrected for multiple testing with the false discovery rate method. Numeric variables are presented in violin plots with single observations depicted as points, and medians with interquartile ranges represented by red diamonds and whiskers. Frequencies for levels of categorical variables are shown in stack plots. Effect sizes and p values are displayed in the plot captions. Numbers of observations in the subsets are indicated in the X axes.*

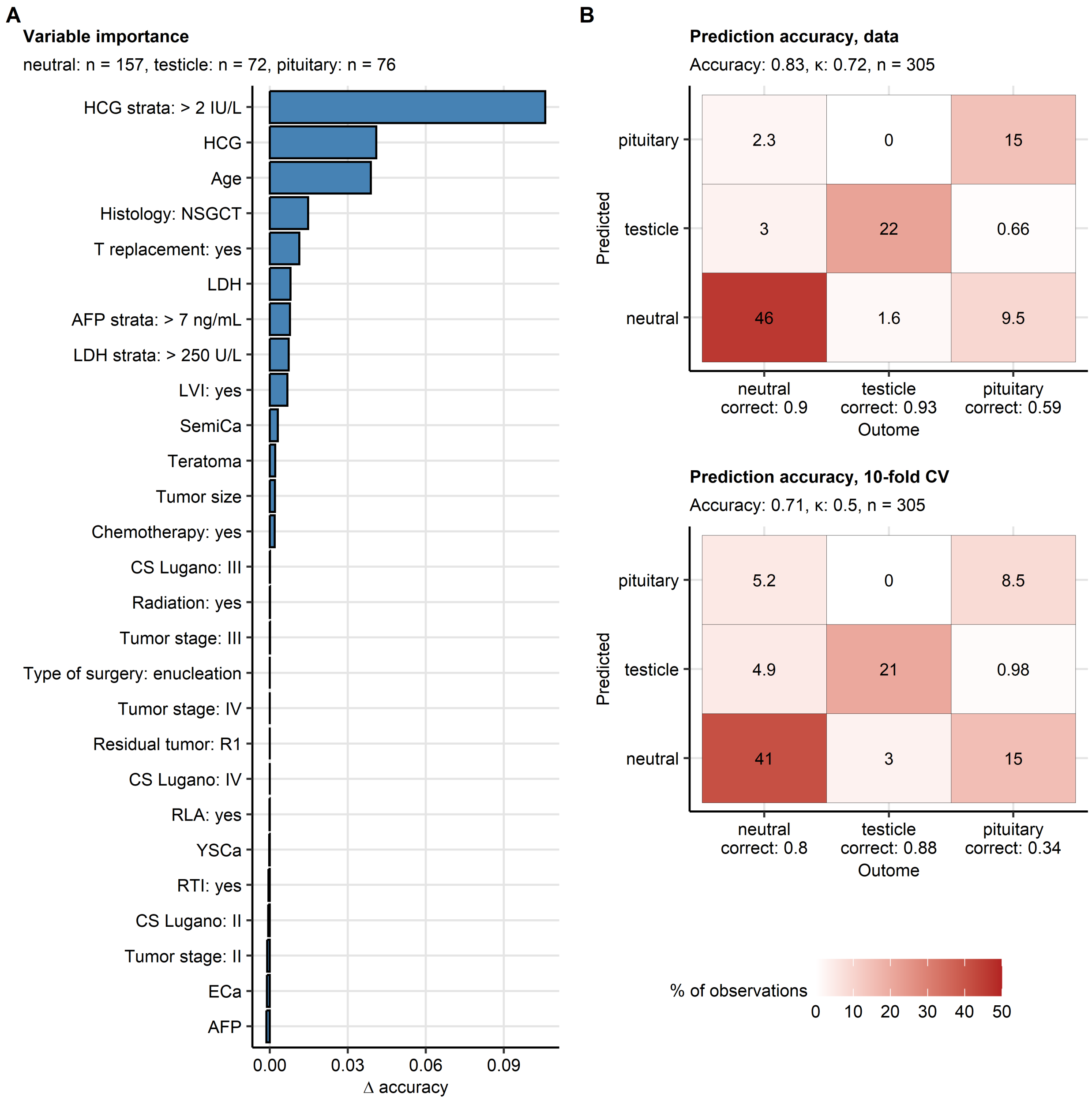

Supplementary Figure S7. Demographic and clinical signature of the hormonal subsets of testicular cancer developed by conditional Random Forest modeling.

Multi-parameter model employing demographic, clinical, pathological, therapy and hormone explanatory variables for discrimination between the hormonal subsets of testicle cancer was developed with the conditional Random Forest algorithm and corroborated by 10-fold cross-validation (CV).

(A) Permutation importance of explanatory variables expressed as difference in accuracy (Δ accuracy) between the original model and the model with a particular variable randomly re-

shuffled. Accuracy differences are displayed in a bar plot. Numbers of samples in the hormonal subsets are shown in the plot caption.

(B) Discriminant performance of the Random Forest model in the original data set and 10-fold CV was visualized as heat maps of confusion matrices. The overall subset assignment accuracy and Cohen's κ are displayed in the plot captions. Fractions of observations correctly assigned to the neutral, testicle and pituitary subsets are indicated in X axes.

HCG: human chorionic gonadotropin; NSGCT: non-seminomatous germ cell tumors; T replacement: testosterone replacement; AFP: alpha fetoprotein; LDH: lactate dehydrogenase; LVI: lymphovascular invasion; SemiCa: percentage of seminoma histology; CS Lugano: Lugano class; RLA: retroperitoneal lymphadenectomy; YSCa: percentage of yolk sac histology; ECa: percentage of embryonic histology; RTI: rete testis invasion.

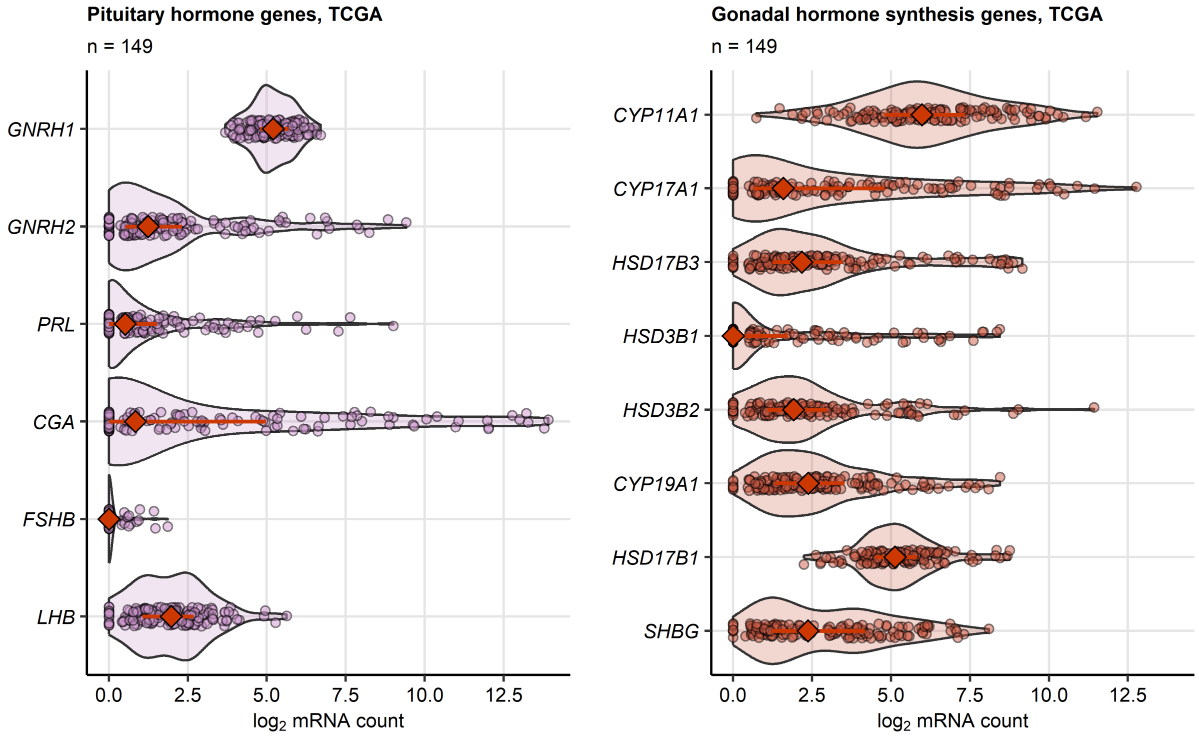

Supplementary Figure S8. Expression of sex hormone-related genes in the malignant tissue in the TCGA testicle cancer cohort.

Expression of pituitary sex hormone genes and gonadal sex hormone-related genes was presented as log_2-transformed transcript counts in violin plots with single cancer samples depicted as points and medians with interquartile ranges depicted as red diamonds with whiskers. Numbers of complete observations are indicated in the plot captions.

References

1. Wickham H, Bryan J, Posit P, Kalicinski M, Komarov V, Leitienne C, Colbert B, Hoerl D, Miller E. readxl: Read Excel Files. (2022) https://cran.r-project.org/web/packages/readxl/index.html

2. Liu J, Lichtenberg T, Hoadley KA, Poisson LM, Lazar AJ, Cherniack AD, Kovatich AJ, Benz CC, Levine DA, Lee AV, et al. An Integrated TCGA Pan-Cancer Clinical Data Resource to Drive High-Quality Survival Outcome Analytics. *Cell* (2018) 173:400–416.e11. doi: 10.1016/J.CELL.2018.02.052

3. Wickham H, Averick M, Bryan J, Chang W, McGowan L, François R, Grolemund G, Hayes A, Henry L, Hester J, et al. Welcome to the Tidyverse. *Journal of Open Source Software* (2019) 4:1686. doi: 10.21105/joss.01686

4. Henry L, Wickham Hadley. rlang: Functions for Base Types and Core R and ’Tidyverse’ Features. (2022) https://cran.r-project.org/web/packages/rlang/index.html

5. Gagolewski M, Tartanus B. Package ’stringi’. (2021) https://cran.r-project.org/web/packages/stringi/index.html http://cran.ism.ac.jp/web/packages/stringi/stringi.pdf

6. Signorell A. DescTools: Tools for Descriptive Statistics. (2022) https://cran.r-project.org/package=DescTools

7. Kassambara A. rstatix: Pipe-Friendly Framework for Basic Statistical Tests. (2021) https://cran.r-project.org/package=rstatix

8. Therneau TM, Grambsch PM. *Modeling Survival Data: Extending the Cox Model*. 1st ed. New York: Springer Verlag (2000).

9. Kassambara A, Kosinski M, Biecek P. survminer: Drawing Survival Curves using ’ggplot2’. (2016) https://cran.r-project.org/package=survminer

10. Croux C, Filzmoser P, Oliveira MR. Algorithms for Projection-Pursuit robust principal component analysis. *Chemometrics and Intelligent Laboratory Systems* (2007) 87:218–225. doi: 10.1016/j.chemolab.2007.01.004

11. Todorov V, Filzmoser P. Comparing classical and robust sparse PCA. *Advances in intelligent systems and computing*. Springer Verlag (2013). p. 283–291 doi: 10.1007/978-3-642-33042-1_31

12. Ripley B. MASS: Support Functions and Datasets for Venables and Ripley’s MASS. (2022) https://cran.r-project.org/package=MASS

13. Bandeen-roche K, Miglioretti DL, Zeger SL, Rathouz PJ. Latent variable regression for multiple discrete outcomes. *Journal of the American Statistical Association* (1997) 92:1375–1386. doi: 10.1080/01621459.1997.10473658

14. Linzer DA, Lewis JB. poLCA: An R package for polytomous variable latent class analysis. *Journal of Statistical Software* (2011) 42:1–29. doi: 10.18637/jss.v042.i10

15. Strobl C, Boulesteix AL, Zeileis A, Hothorn T. Bias in random forest variable importance measures: Illustrations, sources and a solution. *BMC Bioinformatics* (2007) 8:1–21. doi: 10.1186/1471-2105-8-25/FIGURES/11

16. Hothorn T, Hornik K, Zeileis A. Unbiased recursive partitioning: A conditional inference framework. *Journal of Computational and Graphical Statistics* (2006) 15:651–674. doi: 10.1198/106186006X133933

17. Hothorn T, Hornik K, Strobl C, Zeileis A. party: A Laboratory for Recursive Partytioning. (2022) https://cran.r-project.org/web/packages/party/index.html

18. Kuhn M. Building predictive models in R using the caret package. *Journal of Statistical Software* (2008) 28:1–26. doi: 10.18637/jss.v028.i05

19. Wickham Hadley. *ggplot2: Elegant Graphics for Data Analysis*. 1st ed. New York: Springer-Verlag (2016). https://ggplot2.tidyverse.org

20. Wilke CO. *Fundamentals of Data Visualization: A Primer on Making Informative and Compelling Figures*. 1st ed. Sebastopol: O'Reilly Media (2019).

21. Gohel D. flextable: Functions for Tabular Reporting. (2022) https://cran.r-project.org/web/packages/flextable/index.html

22. Allaire J, Xie Y, McPherson J, Luraschi J, Ushey K, Atkins A, Wickham H, Cheng J. rmarkdown: Dynamic Documents for R. (2022) https://cran.r-project.org/web/packages/rmarkdown/index.html

23. Xie Y. *Bookdown: Authoring books and technical documents with R Markdown*. (2016). doi: 10.1201/9781315204963

24. Xie Y. knitr: A General-Purpose Package for Dynamic Report Generation in R. (2022) https://cran.r-project.org/web/packages/knitr/index.html

25. Barnier J. rmdformats: HTML Output Formats and Templates for 'rmarkdown' Documents. (2022) https://cran.r-project.org/web/packages/rmdformats/index.html

26. Benjamini Y, Hochberg Y. Controlling the False Discovery Rate: A Practical and Powerful Approach to Multiple Testing. *Journal of the Royal Statistical Society: Series B (Methodological)* (1995) 57:289–300. doi: 10.1111/j.2517-6161.1995.tb02031.x
